# Mapping Uncertain Risks and Impacts of Substances of Concern in Product Life Cycles - An SSbD Tool for Product Developers

**DOI:** 10.1007/s43615-026-01077-w

**Published:** 2026-08-03

**Authors:** Julieta Bolaños Arriola, Maaike Weber, Nina van Dulmen, Jeroen Guinée, Martina G. Vijver, Ruud Balkenende

**Affiliations:** 1 Faculty of Industrial Design Engineering, Department of Sustainable Design Engineering, Delft University of Technology, Landbergstraat 15, Delft, CE 2628 The Netherlands; 2 Institute of Environmental Sciences (CML), Leiden University, Einsteinweg 2, Leiden, CC 2333 The Netherlands

**Keywords:** Safe and sustainable by design, Product design, Risk assessment, Design method, Substances of concern, Hazardous chemicals

## Abstract

Current Safe and Sustainable by Design (SSbD) frameworks, such as those proposed by the Joint Research Centre, support the development of safer, more sustainable substances, materials, and manufacturing processes. However, these were not designed for product developers or designers and therefore do not align with the iterative and creative nature of product development, nor with its specific needs and constraints. Broad adoption of SSbD requires tiered, resource-efficient assessments tailored to the specific needs of different professionals. The product developer’s perspective is essential for creating safer, more sustainable products, enabling context-specific evaluation of chemical applications, substances of concern (SoC), and product life cycles. In a circular economy, where products, parts, and materials circulate, holistic views become critical, as emissions and exposures may increase and accumulate. Therefore, we developed the Mapping Assessment for Product Substance Safety and Sustainability (MAPSSS) tool, which integrates principles of Life Cycle Assessment (LCA) and Risk Assessment (RA) into a product development context. Through iterative development based on real-life cases of products containing SoC, we combined essential elements of RA and LCA into a product development workflow. Expert input on environmental assessment and ecotoxicology informed each iteration of the process. MAPSSS supports product developers in identifying and assessing SoC risks, trade-offs, uncertainty levels, and environmental impacts across the product life cycle. It enables the identification of risk hotspots in reference products, supporting mitigation strategies and comparison with new solutions. This represents an initial step toward a product-specific SSbD assessment tool for early design stages.

**Supplementary Information:**

The online version contains supplementary material available at 10.1007/s43615-026-01077-w.

## Introduction

Safe and Sustainable by Design (SSbD) aims to be a transformative concept that integrates chemical safety and sustainability throughout the innovation process through continuous assessment, multidisciplinary engagement, and transparency. SSbD is currently approached from a strong chemicals and materials development perspective, focusing on delivering functionality while avoiding harmful impacts on human health and the environment [1–3]. To operationalize the concept of SSbD, available SSbD frameworks integrate life cycle assessment (LCA) and chemical risk assessment (RA) (See e.g., frameworks by the Joint Research Centre (JRC), European Environment Agency (EEA), CEFIC, and TNO [2, 4–6]). Within this approach, RA focuses on identifying hazards and exposures to determine potential adverse effects on human health and the environment, forming the “safety” pillar of SSbD. LCA complements this by providing a standardised, cradle-to-grave evaluation of environmental impacts across all life cycle stages, helping avoid burden shifting and enabling comparisons of alternatives. Together, these elements are combined in an integrated assessment strategy where RA ensures safety, Substances of Concern (SoC) identification guides substitution, and LCA ensures environmental sustainability (see Guinée et al. [7]).

Ideally, the processes involved in implementing the SSbD concept are carried out by interdisciplinary teams. In practice, however, products are often developed outside such environments. Decision-making often falls to product developers, who currently lack the resources and knowledge to conduct intensive safety and sustainability assessments, limiting their ability to make informed choices that effectively mitigate risk.

Products, which we define as physical artefacts designed to fulfil a function or user need directly, are made from materials, and those materials are made of chemical substances. The chemistry hidden inside everyday products can be hazardous. The concept of SoC refers to chemicals with hazardous properties (e.g., persistence, toxicity, bioaccumulation), which can have potentially large-scale impacts that threaten the integrity of Earth system processes, and must be avoided, minimised, or substituted within the SSbD framework to achieve safer material cycles [8, 9]. Many products contain and release SoC throughout their life cycle, generating potential risks to human and environmental health. For illustration, over 60% of the 285 tons of chemical substances produced in Europe in 2022 were classified as hazardous to the environment or human health [10]. Another study from 2025 assessed 16,325 chemicals used in plastics and found that 65.7% of the reported chemicals lacked sufficient data to determine their safety. 25.9% of the chemicals were identified as SoC – hence, only 8.4% could be characterised as safe [11]. In fact, only a small proportion of the chemicals in use have been rigorously assessed for hazards, and even fewer are regulated to limit their use in specific applications [12, 13]. The transition to a circular economy could further aggravate the risks posed by SoCs, as these substances are emitted and accumulate in the environment not only during their initial life cycle but also during subsequent life cycles of products, components, and materials (e.g., through reuse and recycling) [14]. Regulatory involvement and public awareness towards SoC in products and the CE are increasing, as evident from, for example, the European Chemicals Strategy for Sustainability [15] and the requirements for Ecodesign for Sustainable Products Regulation [16], which establishes a framework for ecodesign requirements in products, including the phasing out of substances that hinder circularity, referencing the SSbD criteria as developed by the European Commission.

Current SSbD strategies addressing SoC predominantly focus on the development of inherently safer chemicals and processes (see e.g., van Dijk et al. [17, 18]). However, implementing such approaches is not always feasible [19]. In many cases, the available data remains insufficient to substantiate claims that proposed alternatives offer reduced impacts on human health and the environment [20]. This data insufficiency is particularly pronounced during early stages of substance and product development, where solution spaces are still broad and characterised by high uncertainty [21]. In other words, these complexities and data demands may constrain widespread adoption or lead to a state of indecision [18].

The *product developer’s* perspective plays a crucial role in creating safer, more sustainable products, with around 80% of a product’s environmental impact determined during the design phase [22]. A product perspective allows for context-sensitive considerations related to the specific application of a SoC and the life cycle of the product containing it. For instance, the question of whether certain SoC, such as Perfluoroalkyl and Polyfluoroalkyl Substances (PFAS), are essential may be more appropriately evaluated from a product perspective, which can reveal that specific substances are not always required for particular applications [23, 24]. Furthermore, trade-offs associated with chemical substitution are often only evident when assessed at the product level, preferably considering the full life cycle of the product. For instance, PFAS in synthetic textiles can be replaced with non-fluorinated water-repellent treatments or weaving techniques that achieve waterproofing without additives. However, these alternatives may lead to lower performance, material integrity issues, or functional trade-offs, such as stiffer fabrics unsuitable for specific applications [25, 26]. Furthermore, since a complete characterisation of a SoC is not always feasible, product design can offer a systems-thinking lens to anticipate product-specific conditions and potential behaviours of the SoC. Such an approach also opens possibilities for interventions at multiple levels: material, component, product, system, and process [27]. Despite this potential, the role of product developers in addressing SoC remains limited and is rarely discussed in the scientific literature.

The involvement of product developers, such as industrial designers in design agencies, requires novel approaches and/or tools specific to their expertise, which are currently not available. Alongside the general challenges of SSbD discussed earlier, current frameworks, such as the JRC’s [2], do not adequately serve product developers. Several gaps arise from a product development perspective: The frameworks focus on assessment over ideation, arguing from the chemical/material and its possible applications, not from the product and how its design might or might not make those chemicals essential. In contrast, a product development approach considers the product design itself as a solution space, allowing exploration of design changes at different levels of detail, including specific components within a product system. Such design decisions are often beyond the scope of risk assessment frameworks. This approach can operate in parallel with efforts to create safer chemicals and materials, but can also be used independently by identifying ways to ensure the safe use of chemicals through product design. In other words, existing frameworks do not leverage the iterative, learning-by-doing nature of product development, its strengths in stakeholder management and research in the face of inherent uncertainty, nor its potential to operationalise SSbD holistically rather than through chemical substitution [28]. Furthermore, the necessary material and process safety data are rarely accessible to product developers, due to confidentiality issues along the value chain [29], and are often difficult for non-experts to interpret and manage.

To address this gap, assuming the product developer must tackle all the aforementioned challenges without direct support from other experts, we aim to develop a pragmatic tool that operationalises the principles of LCA and RA in a manner directly applicable to real-world design practice and the product development process. By combining relevant principles from these methods, we aim to build on the increasingly adopted JRC SSbD framework from a product design perspective. Previous research on designing for substance safety, which has identified criteria for developing design tools and methods to address SoC [27, 30, 31], served as a starting point.

This paper presents a first version of the *Mapping Assessment for Product Substance Safety and Sustainability (MAPSSS)* tool for product developers. The current version of the tool supports the targeted risk assessment of suspected or known SoC risks throughout the life cycle of products and their alternatives. Hazard assessment and the identification of all potential SoC in products fall outside of the scope of the current tool, for two separate reasons. Hazard assessment is outside the expertise and sphere of influence of product developers. Although the identification of potential SoC could fall within the expertise and sphere of influence of product design, it remains a challenge due to the lack of value chain transparency and the unavailability of full material declarations. The provision of strategies to deal with the identified SoC risks also falls outside the scope of the tool, as this has been addressed in previous studies [27]. We envision the further development of this tool as an iterative process, in which subsequent research refines the initial version and incorporates new aspects in the future. After outlining its development process, we present the tool, describing its workflow and components, and illustrate its use with a case study. The paper focuses on describing the assessment tool and does not provide recommendations for potential design solutions to SoC risks.

## Method – Tool Development Process

To develop the MAPSSS tool, we integrated key elements of RA and LCA into a product development workflow. The current version was created by adapting LCA and RA principles, drawing on theoretical foundations, expert collaboration, and repeated testing on two case studies that informed the design and refinement of its components. The tool has been developed through a learning-based approach, and we envision its continued evolution as an iterative and incremental process, in which ongoing research will further refine this initial version and incorporate new functionalities over time. In this section, we present: (1) tool development criteria, (2) case selection and iterative development process of the tool, (3) adaptation of extracted LCA elements for the tool, (4) adaptation of extracted RA elements for the tool, and (5) tool integration into the product development process.

### Criteria for the Development of the Tool

We built on previous research that explored approaches and strategies for product developers to address the risks of SoC in products. Bolaños Arriola et al. [30] established requirements for the development of tools and methods that support product developers in mitigating SoC risks and minimising the overall footprint of chemical, material, and product life cycles. Based on this research, we defined the following criteria for the development of the tool:

1. Support the identification and assessment of SoC risks throughout the product life cycle by a product developer.
2. Support the identification of knowledge gaps and uncertainty by a product developer.
3. Support decision-making under uncertainty by a product developer.
4. Support the comparison of product design alternatives, regarding SoC risks and potential environmental trade-offs over the life cycle.
5. Integrate into the product development process, adopting an iterative approach and a life cycle perspective.
6. Consider potential data limitations and the disciplinary boundaries of product development expertise while leveraging the strengths of design practice, such as problem framing, systems thinking, and stakeholder involvement.

The remainder of the method section describes the approach for fulfilling these criteria.

### Selection and Iterative Analysis of Cases for Tool Development

To develop the tool, we analysed and repeatedly applied versions of the MAPSSS tool to two different cases of products containing SoC for which alternative options exist. For each case, we considered a non-exhaustive list of previously implemented risk mitigation strategies (hereafter referred to as alternatives) (Table 1). The development process of the tool involved adapting elements of RA and LCA to create life cycle maps for both the reference products and their respective alternatives, identifying and prioritising SoC risk hotspots throughout the product life cycle, and evaluating potential trade-offs. The iterative process of mapping the cases helped refine the adapted RA and LCA elements, define the tool steps, and improve visualisation features. The two different cases allowed us to develop a versatile tool applicable to a wide range of product types, containing several SoC that behave differently, resulting in various possible risk characterisation scenarios.

**Table 1:** The table presents the selected cases for the analysis and iterative development of the MAPSSS tool. Design alternatives are listed for each case, along with the corresponding design strategies according to Bolaños Arriola et al. [27]

Selected product and SoC (reference product)	Selected alternatives	Design strategy (Risk mitigation strategy)
Polyurethane foam mattresses containing hazardous flame retardant, Tris(chloropropyl) phosphate (TCPP).	Polyester 3D structure that is inherently flame-retardant replaces the foam.	Avoid: Alternative function
	Denser foam with improved fire retardancy replaces the low-density foam; the flame-retardant content is reduced and replaced by melamine.	Reduce: Reduce SoC content Avoid: Substitution
	Closed-loop recycled foam.	Control: Controlled recovery
Agricultural mulch film generating microplastics. Polyethylene (PE) film (non-biodegradable), no recovery.	Polybutylene adipate terephthalate (PBAT) film (biodegradable) onsite degradation.	Avoid: Substitution
	PE film (non-biodegradable) with recovery.	Control: Controlled recovery

The two cases were selected based on the following criteria:

- The cases contain different SoC types with different emission mechanisms and hazard profiles, as defined in the introduction.
- The cases have diverse exposed populations and use patterns, resulting in a variety of risk hotspots.
- The cases include different design alternatives/strategies aimed at mitigating the SoC.

To describe the design alternatives, we used the framework by Bolaños Arriola et al. [27] which defines three categories of design strategies to address SoC in products: Avoid (any intervention that prevents the use of the SoC, for example, substitution and alternative value propositions), Reduce (any intervention that lowers the content of a SoC in a product, reducing the chances of exposure, or the potential accumulation of a SoC ), and Control (any intervention that prevents emissions of, or exposure to a SoC throughout a product’s lifecycle, for example, designs that allow the controlled recovery of the SoC). Collectively, the cases illustrate several of these strategies, as shown in Table 1.

Both cases were analysed using the following protocol:

1. A life cycle map indicating exposure scenarios and risk hotspots was made for the reference product.
2. A life cycle map indicating exposure scenarios and risk hotspots was made for each of the selected alternatives.
3. The maps of the reference products were compared to those of their corresponding alternatives to identify potential risks and environmental trade-offs.

Data on the cases was collected through desk research as well as personal correspondence with manufacturers and other stakeholders throughout the value chain (product developers, recyclers, environmental researchers and a standards developer). The mapping process was conducted independently by three researchers, following the protocol. The results were reviewed collaboratively to refine the tool elements and ensure alignment with the previously established criteria. Additionally, RA and LCA experts were consulted to revise the resulting maps and assess the tool’s alignment with the established criteria. All resulting maps are available in Supplementary Material A. The mattress containing flame retardants is used as a case study in section 3.3 to illustrate the tool and its components.

### Adaptation of LCA Elements for Product Developers

We extracted elements of LCA and adapted them to meet the criteria introduced in section 2.1. We did this based on existing LCA guides that apply the method in accordance with ISO standards, such as the Handbook of LCA [32]. Three LCA elements were extracted and adapted to fulfil the criteria presented in section 2.1 (specifically C2, C4, and C5):

1. Visual elements of *lifecycle mapping* or *system mapping* to represent the life cycle of the product, including unit processes, inflows and outflows, and a system boundary.
2. The definition of a functional unit and reference flows to enable the comparison of the reference product to its alternatives.
3. We developed a set of what we defined as *trade-off* *rules* to perform as a simplification and combination of the inventory analysis and interpretation steps of LCA. We did this by characterising typical trade-offs associated with design interventions at the chemical, material, component, product, and system levels. For this, we incorporated the same risk mitigation strategies introduced in section 2.2 (Avoid, Reduce, Control) [27]. We organised the resulting rules by the life cycle stage they address, resulting in an initial list supported by examples found in literature. These rules support the identification of environmental impacts that may emerge when chemical risks are mitigated through redesign across the product life cycle.
4. Since this is a high-level approach to estimating potential environmental trade-offs, the trade-off rules currently only consider climate change and resource use as environmental impact categories.

The adaptation of these LCA elements was refined through the iterative process presented in section 2.2 and by incorporating expert feedback.

### Adaptation of RA Elements for Product Developers

We extracted elements of chemical risk assessment and adapted them to meet the established criteria. Our adaptation aligns with established RA frameworks [7, 33, 34]. We adapted these frameworks to facilitate a combined qualitative and semi-quantitative identification and prioritisation of SoC risks to human and ecosystem health throughout the life cycle of products or their alternatives. Previous research has identified the possibility and highlighted the value of conducting preliminary safety evaluations in the early stages of development, despite data limitations [35]. Similarly, given data availability limitations in the early stages of development, the JRC also recommends a tiered approach to the assessment [36]. Chemical risk assessment is inherently adaptable and can be tailored to fit an intended purpose, considering the nature and lack of available data, as well as practical constraints such as limited access or knowledge in the tool user. To this end, we followed the recommendations of the Swedish Chemical Agency [37] on how to assess risk scenarios qualitatively in case of uncertainty or data scarcity. As such, we developed a framework that follows a similar structure to the original, but we excluded RA elements for which data may not be available to product developers, or are outside their expertise, such as hazard identification and deriving predicted no-effect concentrations.

We identified and reformulated the necessary variables for product developers to describe and assess risk scenarios based on the effects, characteristics, usage patterns, and behaviour of a substance throughout the product’s life cycle. Throughout the iterative process of mapping the cases, we developed visualisation elements for product developers to describe exposure scenarios relevant to human and ecosystem health using the reformulated variables. These include, but are not limited to:

- Hazard category of the substance.
- Volume of the emitted substance.
- Use pattern and exposed population.
- Use conditions and contextual factors.
- Risk mitigation measures.

Using those specific variables, we also formulated a scoring system for product developers to assign values to the identified risk scenarios, use the results to identify potential risk hotspots, and facilitate comparison. This scoring system calculates what we refer to as the *concern score*. We defined options from which product developers can choose to describe and assess each of the defined variables. Each option was assigned a value to express its contribution to risk, translating the qualitative assessment into quantitative indicators. These values were assigned based on existing RA theory, available information on SoC, and input from RA experts. The rationale behind the values assigned for each variable is presented in Supplementary Material B. The adaptation of these RA elements was refined through the iterative process described in section 2.2 and through expert feedback.

In addition to the concern score calculator, we also developed an uncertainty calculator, which allows product developers to indicate the level of uncertainty (low, medium, or high) for each of the concern score variables. This addition meets the criteria points C2 and C6 by facilitating estimation when data are limited, providing an indication of the assessment’s reliability, and highlighting data gaps. Finally, to meet criteria C3 and C4, we developed a set of interpretation guidelines for product developers to (a) identify risk hotspots, (b) gain awareness of uncertainty, and (c) compare alternatives to the reference product, using the resulting maps.

### Integration with Product Development Workflow

Finally, to establish the necessary steps of the MAPSSS tool in accordance with product development practices, we defined a workflow by integrating the tool’s intended use into the double diamond design process model [38]. This model was selected over, for example, the stage-gate model, as it represents the exploratory and converging nature of the product development process, implying the type of iterative process that we want to support. The model outlines the four main phases of the product development process: discover, define, develop, and deliver.

We defined the tool’s workflow by establishing tool stages containing steps that correspond to each phase of the product development process: for the *Discover phase*, steps are set to explore the current problems in the reference product, for the *Define phase*, steps are set to identify and define risk hotspots in the reference product, for the *Develop and Deliver* phases, steps are set to explore alternatives, while identifying their effects on SoC risks, potential trade-offs, and to compare results to inform decision making for further development.

## Results – The Mapping Assessment for Product Substance Safety and Sustainability Tool

This section presents the tool resulting from the criteria and development process described in the Method section. We first present a detailed description of all components and functions of the tool (Section 3.1). These include: elements to describe and visualise SoC risks across the product lifecycle, calculators for concern and uncertainty scores, interpretation and comparison guidelines, and trade-off rules to identify potential trade-offs. We then present the proposed set of steps for applying the tool and integrating it into the design process (Section 3.2). Finally, we illustrate the use of the tool with an example case: hospital mattresses containing toxic flame retardants (FR), which we refer to as the reference product (Section 3.3). We present the resulting map of the reference product and maps for two alternatives used to mitigate risks posed by flame retardants. We emphasise that the use of this case is for illustrative purposes only. The complete set of resulting maps of the mattress case and the mulch film case is available as Supplementary Material A.

### Tool Components and Functions

Below, we provide a detailed description of the resulting components and functions of the MAPSSS tool. These are a result of the iterative mapping process and adaptation of RA and LCA elements, as described in the method section. A protocol document guiding product developers in applying the components is available in Supplementary Material D.

#### Elements to Describe and Visualise SoC Risks in the Product Lifecycle

The tool provides a set of mapping elements for the product developer to describe the product system, the SoC’s presence throughout the product’s lifecycle, and all identified exposure scenarios. These elements are used both to map the reference product and its potential design alternatives. These resources support users with limited LCA and RA experience, promoting the standardisation of maps and enabling comparison across cases. The mapping elements are built upon ISO standards for LCA, as elaborated by Guinée et al. [32], and existing RA guidelines [34, 39].

**Fig. 1:**
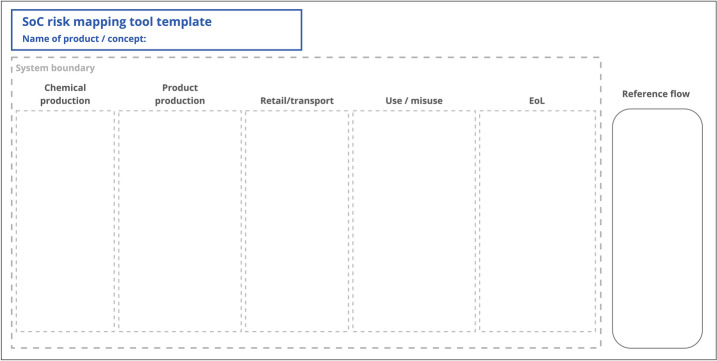
The life cycle template has five predefined lifecycle stages. The template serves as a basis for system and exposure scenario mapping and presents the reference flow of the analysed product or alternative

**Fig. 2:**
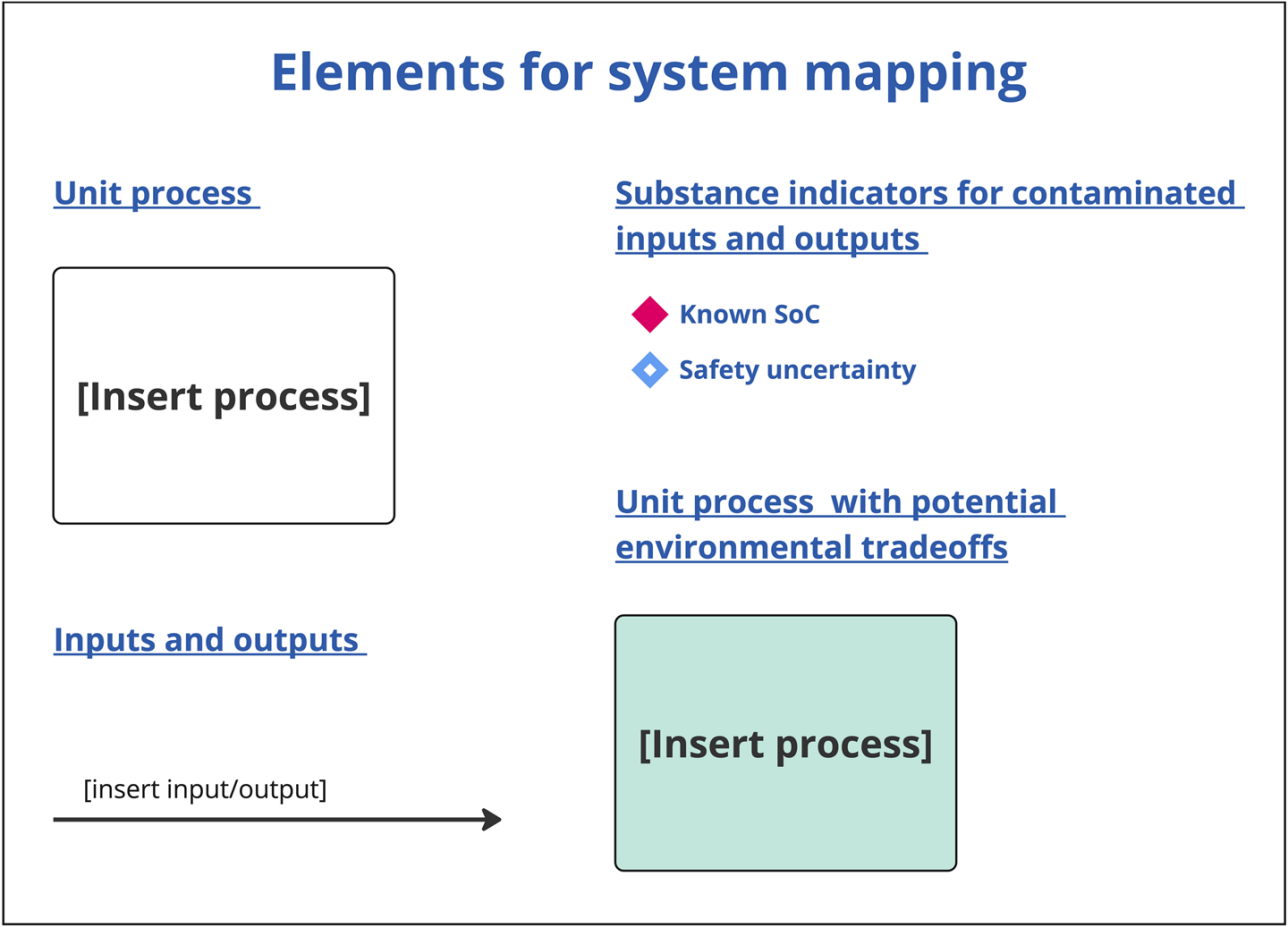
Visual elements for system mapping, indicators for “contaminated” inputs and outputs, and indicators for potential environmental trade-offs

**Fig. 3:**
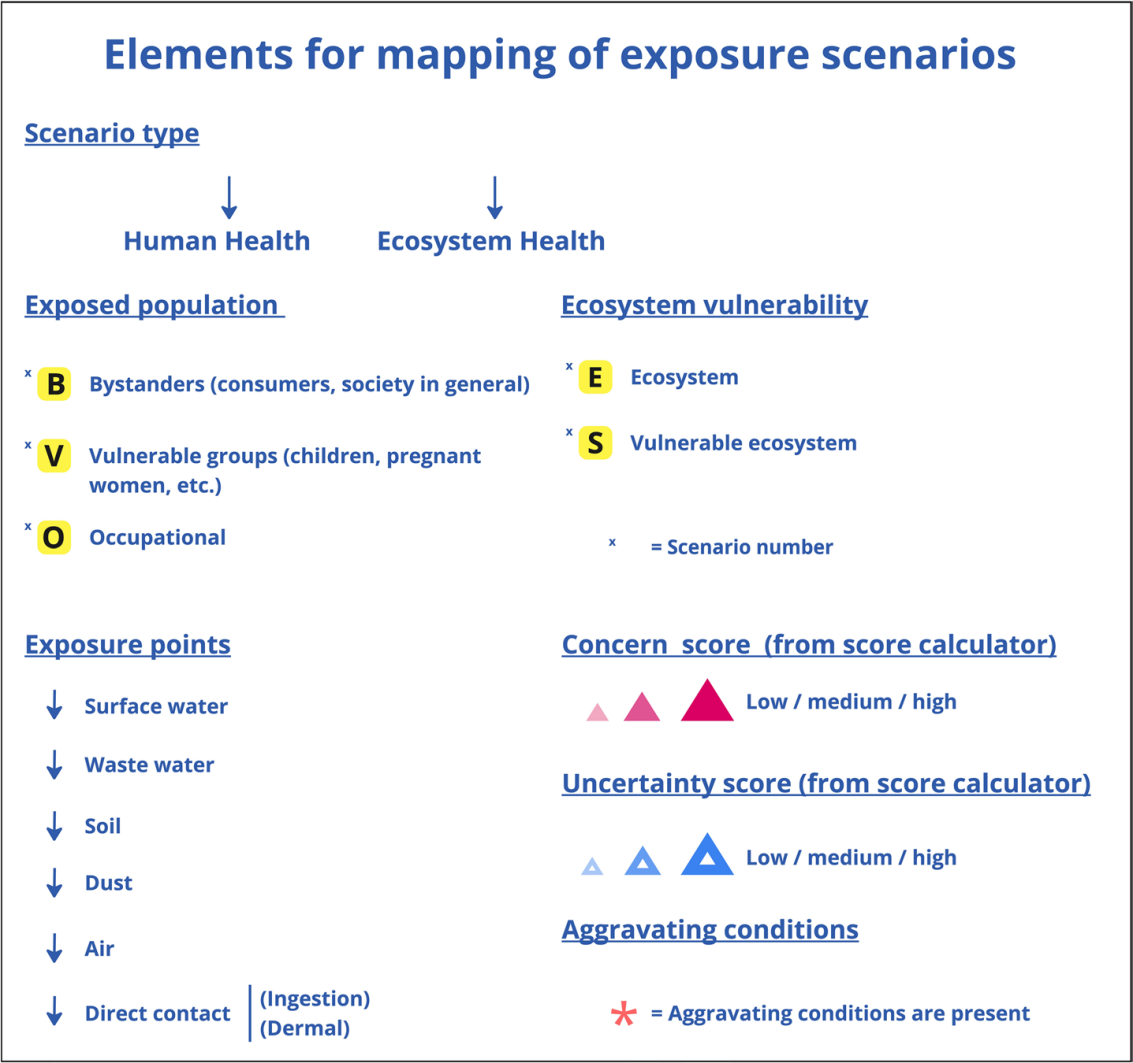
Visual elements to map and describe exposure scenarios, indicating whether human health or environmental health is affected (scenario type), the exposed groups (exposed population or ecosystem vulnerability), exposure points, potential presence of aggravating conditions (e.g., heat, misuse, etc.), and resulting concern and uncertainty scores

First, the tool provides a lifecycle template (Fig. 1) that serves as a basis for all other visual elements (Figs. 2 and 3). The template requires product developers to define a functional unit for comparing different product alternatives and to indicate the reference flow for assessing a specific alternative (Fig. 1). The template predefines five life cycle stages:

- Chemical production: all processes required to produce the analysed substance and other relevant substances in materials.
- Product manufacturing: material production, processing, and assembly.
- Retail: processes required to deliver the product to its use.
- Use: activities related to product operation and maintenance, including potential misuse.
- End-of-life (EoL): processes to destroy, repurpose, or reprocess the used product.

The elements provided in Fig. 2 can be used to map relevant unit processes, along with their inputs and outputs, considering the system boundary (e.g., waste generated during production can be indicated as an output that leaves the system boundary). Processes should be described in as much detail as possible, considering contextual conditions (e.g., location), to enhance the accuracy of the analysis. Initial assessments based on approximations are possible, since the level of detail can be increased as more data becomes available during product development (e.g., bill of materials), through stakeholder collaboration, consultation of LCI databases, or expert consultation. The visualisation of circular scenarios is supported whenever applicable.

**Fig. 4:**
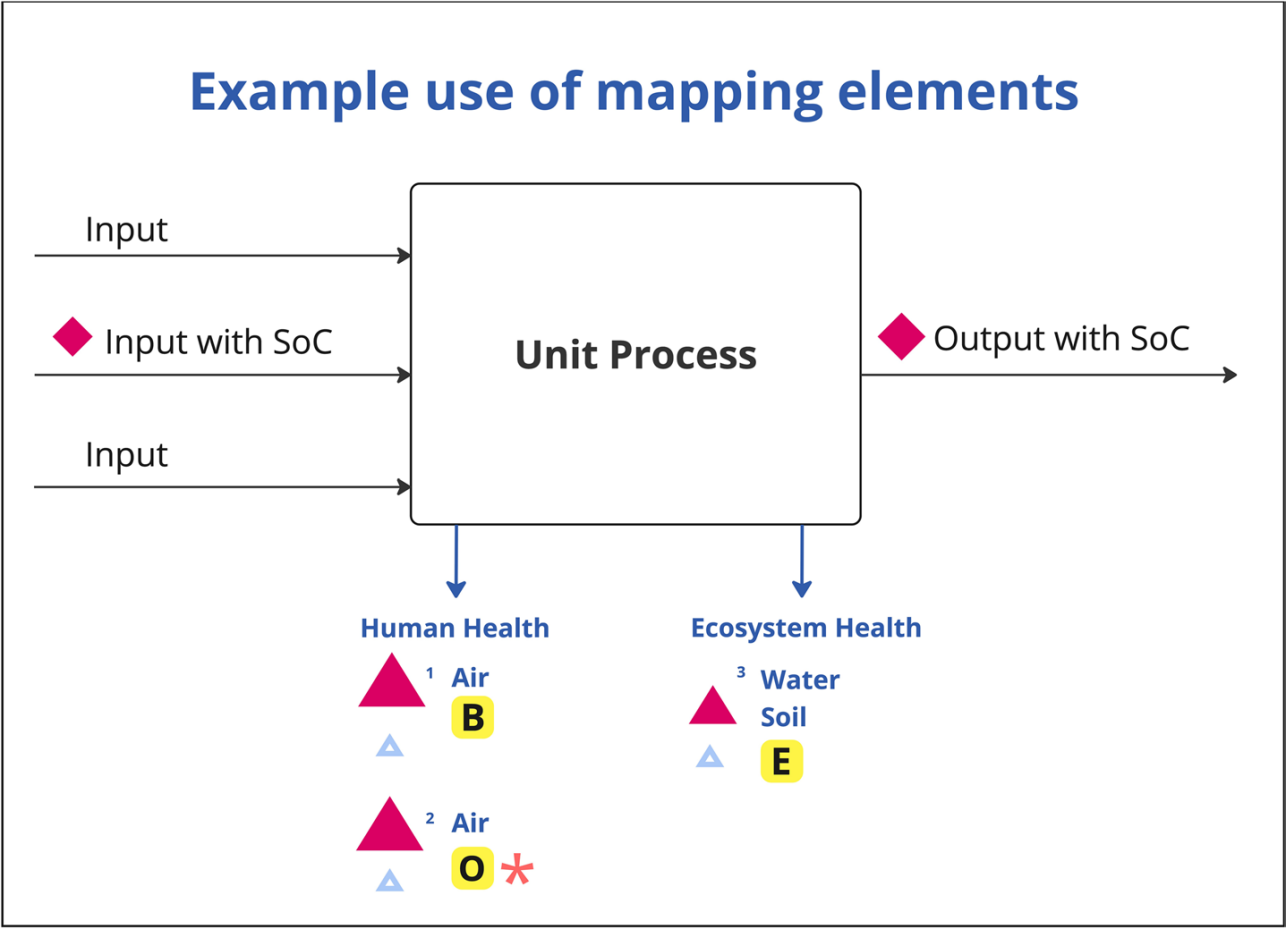
Proposed convention to apply the tool’s mapping elements. The example depicts a unit process with a contaminated input and output, and describes three exposure scenarios, two for human health via air to bystanders and occupational, and one for ecosystem health via water and soil. Aggravating conditions appear to be present for the occupational exposure scenario

The tool also supports mapping the presence of SoC across the product life cycle. This is done by placing rhomboid markers (Fig. 2) on the inputs and outputs where the SoC is expected to be present. This indicates the behaviour of the SoC and identifies “contaminated” inputs and outputs. In products where circular economy strategies are implemented, potentially contaminated outputs can flow back into the system (in closed-loop circularity) or out of it (in open-loop circularity). The pink (filled) marker identifies substances that are known as SoC. Conversely, the blue (outline) marker highlights substances with unknown or inconclusive safety profiles. This helps product developers determine which processes may be of concern and which are already addressed by mitigation strategies. In addition, these indicators flag potential risks associated with outputs crossing the system boundary, as their fate is unknown and their impacts can therefore not be assessed. Figure 4 illustrates an example of the proposed convention for utilising the described mapping elements (Figs. 2 and 3).

The tool provides a system for identifying and describing exposure scenarios throughout the product’s life cycle (Fig. 3). Exposure scenarios are defined by three variables: whether humans and/or ecosystems are impacted, the points at which exposure can occur (e.g., soil, water), and the exposed population (e.g., occupational, bystanders, vulnerable ecosystems) (Fig. 3).

Product developers can define multiple exposure scenarios per unit process as indicated in the example in Fig. 4. For human health, a scenario is created for each exposed population; for ecosystem health, one scenario is considered per unit process. The example in Fig. 4 illustrates three scenarios: two for human health (both via air, affecting two distinct groups: bystanders and workers) and one for ecosystem health (via water and soil). An additional specification (pink asterisk) can be added if aggravating conditions are suspected, e.g., temperature or humidity variation, wear and tear from behaviour or machinery, or interaction with other substances during maintenance. Product developers should assess whether design-specific conditions (e.g., use at high temperatures, shredding at end of life) combined with intrinsic substance characteristics (e.g., solubility, volatility, adsorption) may aggravate emissions. With these elements, the tool enables product developers to consider a variety of product-specific scenarios, including relevant stakeholders and other factors such as contextual factors and behaviour.

#### Concern and Uncertainty Score Calculator

**Fig. 5:**
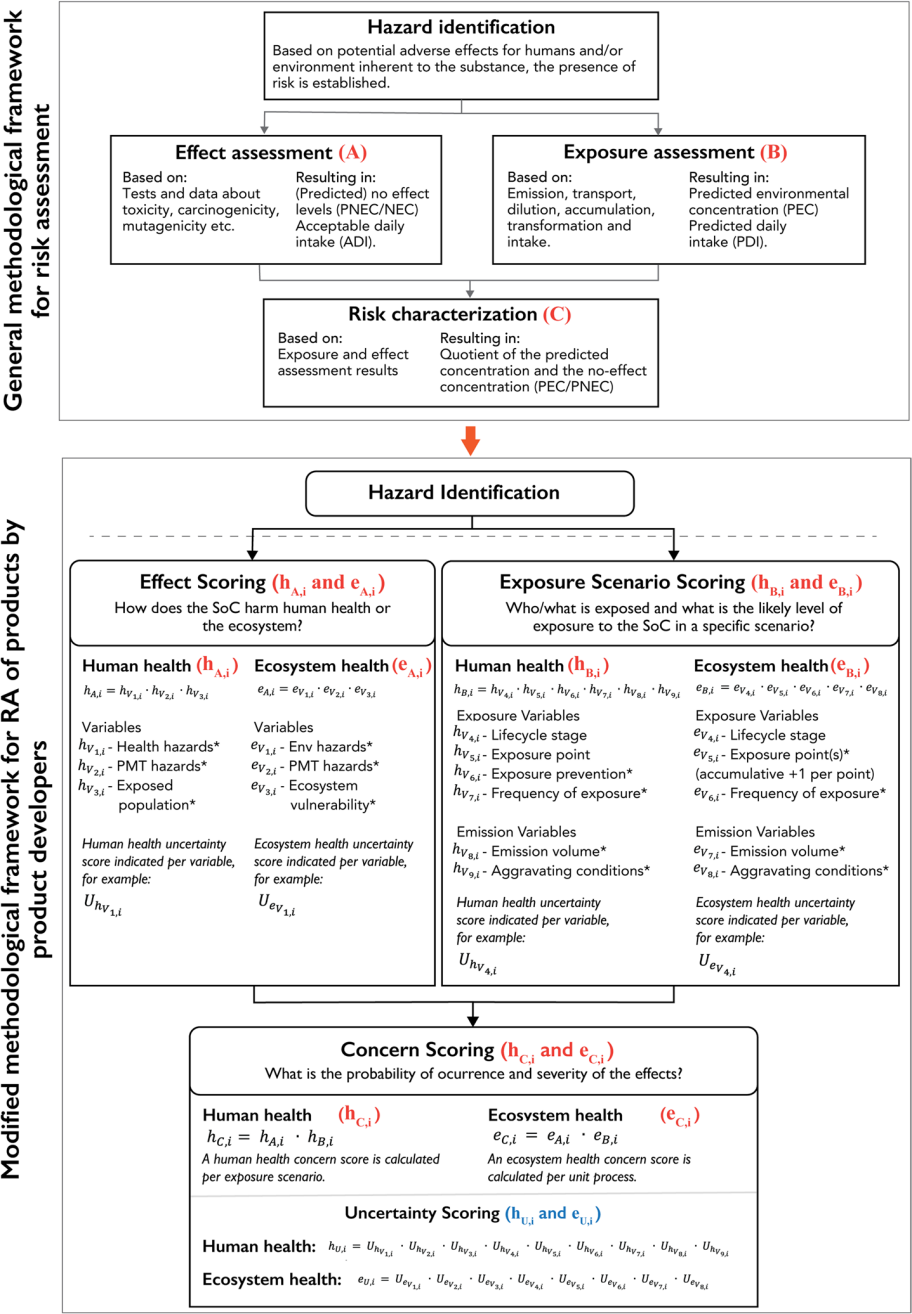
Overview of how the methodological RA framework (as described, for example, by Boersema & Reijnders [33]) was modified for product developer use. The top panel depicts the RA framework, and the bottom panel depicts the modified approach to calculate the concern score, including all considered variables to assess human health and ecosystem health-related risks. Variables marked with a star weight the concern score calculation, while those without it are only descriptive. The concern, effect, and exposure scenario scores are highlighted in red, while all uncertainty scores are highlighted in blue

The exposure scenarios defined on the map can be assessed and scored to identify hotspots within the product life cycle. To achieve this, the tool offers a concern score calculator, a methodological adaptation and simplification of the RA framework. Figure 5 illustrates how the RA framework was adapted for product developers, as well as the variables used to calculate the concern score.

The original RA framework was adapted in accordance with the predefined criteria (Section 2.1) to meet the product developer’s needs [30], taking into account their workflows, the scope of their capabilities, and the resources [30] available to them when assessing chemical-related risks. The tool, therefore, excludes quantitative forms of assessment that rely on complex inputs or data obtained from lab and field tests. Through a semi-quantitative approach, product developers can translate qualitative information on the characteristics and behaviour of the substance throughout the product’s life cycle into semi-quantitative scores. The scoring system is based on predefined variables that include options to describe and assess exposure scenarios, with preassigned weights (Tables 2 and 3). Currently, the concern score calculator is made available to product developers as an Excel template (Supplementary material C), which incorporates all elements and calculations described below. In this section, we provide a detailed explanation of the working principles of the concern score calculator and the assigned variables. A complete explanation of the adaptation of all RA elements and the rationale behind the assigned values is available as Supplementary Material B.

**Table 2:** Variables to calculate the effect score ($\:{h}_{A,\:i\:}$and$\:{e}_{A,\:i\:}$) as indicated in Fig. 5. Each variable has predefined options that the product developer can select from to describe and assess them. Each option has an assigned weight (points). For example, if the variable ${e}_{{{V}_{1,i}}_{}}$Hazard category is marked as toxic to the aquatic environment, then ${e}_{{{V}_{1,i}}_{}}$= 4pts

Human health Effect scoring (${{h}}_{{A},\:{i}\:}$),$\:{h}_{A,\:i}={h}_{{V}_{1,i}}\bullet\:\:{h}_{{V}_{2,i}}\bullet\:{h}_{{{V}_{3,i}}_{\:}}$			Ecosystem health Effect scoring (${{e}}_{{A},\:{i}\:}$),$\:{e}_{A,\:i\:}={e}_{{{V}_{1,i}}_{\:\:}}\bullet\:{e}_{{V}_{2,i}}\bullet\:{e}_{{V}_{3,i}}$		
Variable	Options to describe variable	Weight (pts)	Variable	Options to describe variable	Weight (pts)
*(* $\:{{h}}_{{{{V}}_{1,{i}}}_{\:}}$ *)* *Hazard category*	A. Systemic health hazards: Carcinogenic, mutagenic or toxic to reproduction (CMR)	*4*	(${{e}}_{{{V}}_{1,\boldsymbol{i}}}$*)* *Hazard category*	A. Toxic to aquatic environment	4
	B. Physical hazards (explosive, corrosive, flammable, gas under pressure, oxidising)	2		B. Damage to the ozone layer	2
	C. Direct health hazards: acute toxicity, specific organ toxicity, skin corrosion, skin or respiratory sensitisation, eye damage	2			
*(* $\:{{h}}_{{{{V}}_{2,{i}}}_{\:}}$ *) Persistence / mobility / bioacc.*	A. Very persistent and very bioaccumulating or Very Persistent, very Mobile (vPvB or vPvM)	4	*(* ${{e}}_{{{V}}_{2,{i}}}$ *) Persistence / mobility / bioacc.*	A. Very persistent and very bioaccumulating or Very Persistent, very Mobile (vPvB or vPvM)	4
	B. Persistent, bioaccumulating or mobile, and toxic (PBT or PMT)	3		B. Persistent, bioaccumulating or mobile, and toxic (PBT or PMT)	3
	C. Not applicable	1		C. Not applicable	1
*(* ${{h}}_{{{{V}}_{3,{i}}}_{\:}}$ *)* *Exposed population*	A. Vulnerable groups (children, pregnant, etc.)	*3*	*(* ${{e}}_{{{V}}_{3,\boldsymbol{i}}}$ *) Ecosystem vulnerability*	A. Vulnerable ecosystem	3
	B. People (consumers, society)	1.5		B. Ecosystem	1.5
	C. Occupational	1.5			

In traditional RA, chemical hazard identification aims to recognise and document all chemicals that could cause harm to people, property, or the environment. It is done based on chemical data, through analysis of lab and in situ data. For this tool, we assume that the SoC and its hazards have been previously identified through bill of materials (BOM) analysis, material safety data sheets, and available hazard data. This hazard identification element of risk assessment is thus not explicitly supported by the tool.

The concern score calculator follows two different pathways to support the assessment of scenarios that impact human health and scenarios that impact ecosystem health. In Fig. 5, we observe that a human health concern score ($\:{h}_{C,\:i\:}$) or an ecosystem health concern score ($\:{e}_{C,\:i\:}$) corresponds to the risk characterisation step in the RA framework. In this formula, the *i* refers to a particular SoC. Concern scores should be calculated for each of the exposure scenarios previously defined by the product developer in the map. A concern score ($\:{h}_{C,\:i\:}$ and $\:{e}_{C,\:i\:}$) is obtained by multiplying the results from the Effect score ($\:{h}_{A,\:i\:}$ and $\:{e}_{A,\:i\:}$) by the results obtained from the Exposure score ($\:{h}_{B,\:i\:}$ and $\:{e}_{B,\:i\:}$):

1 $$\begin{array}{c}{h}_{C,\:i\:}=\:{h}_{A,\:i\:}\:\bullet\:\:{h}_{B,\:i\:}\end{array}$$

and

2 $$\begin{array}{c}{e}_{C,\:i\:}=\:{e}_{A,\:i\:}\:\bullet\:\:{e}_{B,\:i\:}\end{array}$$

The effect score ($\:{h}_{A,\:i\:}$ and $\:{e}_{A,\:i\:}$), which is equivalent to the effect assessment step in the RA framework, is calculated by multiplying three variables (Fig. 5; Table 2). In case of a human health scenario, the human health hazard category of the substance ($\:{h}_{{{V}_{1,i}}_{\:}}$), its persistence/mobility/accumulation characteristics ($\:{h}_{{{V}_{2,i}}_{\:}}$), and the exposed population ($\:{h}_{{{V}_{3,i}}_{\:}}$) determine the effect score ($\:{h}_{A,\:i\:}$). If ecosystem health is affected, the ecosystem health hazard category of the substance ($\:{e}_{{{V}_{1,i}}_{\:}}$), its persistence/mobility/accumulation characteristics ($\:{e}_{{{V}_{2,i}}_{\:}}$), and the ecosystem vulnerability ($\:{e}_{{{V}_{3,i}}_{\:}}$) determine the effect score ($\:{e}_{A,\:i\:}$).

The exposure score ($\:{h}_{B,\:i\:}$ and $\:{e}_{B,\:i\:}$), which is equivalent to the exposure assessment step in the RA framework, is calculated by multiplying several variables (Fig. 5; Table 3). In the case of a human health scenario, the exposure score ($\:{h}_{B,\:i\:}$) considers four variables describing exposure ($\:{h}_{{{V}_{4,i}}_{\:}}$ to $\:{h}_{{{V}_{7,i}}_{\:}}$) and two variables describing emissions ($\:{h}_{{{V}_{8,i}}_{\:}}$ and $\:{h}_{{{V}_{9,i}}_{\:}}$) (Table 3). In the case of an ecosystem health scenario, the exposure score ($\:{e}_{B,\:i\:}$) considers three variables describing exposure ($\:{e}_{{{V}_{4,i}}_{\:}}$ to $\:{e}_{{{V}_{6,i}}_{\:}}$), and two describing emissions ($\:{e}_{{{V}_{7,i}}_{\:}}$ and $\:{e}_{{{V}_{8,i}}_{\:}}$) (Table 3).

**Table 3:** Variables to calculate the exposure scenario score ($\:{h}_{B,\:i\:}$and$\:{e}_{B,\:i\:}$) as indicated in Fig. 5. Each variable has predefined options that the product developer can select from to describe and assess them. Each option has an assigned weight (points). For example, if the variable$\:{e}_{{V}_{6,i}}$Frequency of exposure is marked as chronic, then$\:{e}_{{V}_{6,i}}$= 2.5 points. Variable$\:{e}_{{V}_{5,\:i}}$gets an accumulative weight of + 1 for each selected exposure point (e.g., Air + Wastewater = 2). Variables marked as descriptive have no assigned weight as they characterize the scenario but have no influence on the resulting concern score ($\:{h}_{C,\:i\:}$and$\:{e}_{C,\:i\:}$). The variable Exposure prevention is, in this case, not applicable to ecosystem health

	Human health exposure scoring (${{h}}_{{B},\:{i}}$), $\:{h}_{B,\:i\:}={h}_{{V}_{4,i}}\bullet\:{h}_{{V}_{5,i}}\bullet\:{h}_{{{V}_{6,i}}_{\:}}\bullet\:{h}_{{{V}_{7,i}}_{\:}}\bullet\:{h}_{{{V}_{8,i}}_{\:}}\bullet\:{h}_{{{V}_{9,i}}_{\:}}$			Ecosystem health exposure scoring (${{e}}_{{B},\:{i}\:}$), $\:{e}_{B,\:i\:}=\:{e}_{{V}_{4,i}}\bullet\:{e}_{{V}_{5,\:i}}\bullet\:{e}_{{V}_{6,i}}\bullet\:{e}_{{V}_{7,i}}\bullet\:{e}_{{{V}_{8,i}}_{\:}}$		
	*Variable*	Options to describe variable	*Weight* *(pts)*	*Variable*	Options to describe variable	*Weight* *(pts)*
Exposure description variables	***(*** ${{h}}_{{{V}}_{4,{i}}}$ ***)*** ***Life cycle stage*** *(descriptive)*	Life cycle stage and unit process in which the scenario occurs		***(*** ${{e}}_{{{V}}_{4,{i}}}$ ***)*** ***Life cycle stage*** *(descriptive)*	Life cycle stage and unit process in which the scenario occurs	
	***(*** ${{h}}_{{{V}}_{5,{i}}}$ ***)*** ***Exposure point*** *(descriptive)*	Point in which a population comes in contact with the substance. Air, wastewater, surface water, soil, dust, direct contact, (ingestion or dermal)		**(** ${{e}}_{{{V}}_{5,{i}}}$ **)** ***Exposure point.*** *Several can be selected. The weight increases according to the number of selected points (accumulative)*	A. Air	+ 1
					B. Wastewater	+ 1
					C. Surface water	+ 1
					D. Soil	+ 1
					E. Dust	+ 1
	***(*** ${{h}}_{{{V}}_{6,{i}}}$ ***)*** ***Exposure prevention***	A. Not in place	1			
		B. In place and exposure is reduced	0.5			
		C. In place and exposure is eliminated	0			
	***(*** ${{h}}_{{{V}}_{7,{i}}}$ ***)*** ***Frequency of exposure***	A. Chronic	2.5	***(*** ${{e}}_{{{V}}_{6,{i}}}$ ***)*** ***Frequency of exposure***	A. Chronic	2.5
		B. Repeated	2		B. Repeated	2
		C. Single	1.5		C. Single	1.5
Emission description variables	***(*** ${{h}}_{{{V}}_{8,{i}}}$ ***)*** ***Emission volume*** *(According to risk management measures that reduce emissions)*	A. No control mechanism in place - emissions occur	1	***(*** ${{e}}_{{{V}}_{7,{i}}}$ ***)*** ***Emission volume*** *(According to risk management measures that reduce emissions)*	A. No control mechanism in place - emissions occur	1
		B. Control mechanism in place – reduced emissions	0.5		B. Control mechanism in place – reduced emissions	0.5
		C. Control mechanism in place – no emissions occur	0		C. Control mechanism in place – no emissions occur	0
		D. Known concentrations and/or emissions remain below threshold values	0		D. Known concentrations and/or emissions remain below threshold values	0
	***(*** ${{h}}_{{{V}}_{9,{i}}}$ ***)*** ***Aggravating conditions***	A. No aggravating conditions	1	***(*** ${{e}}_{{{V}}_{8,{i}}}$ ***)*** ***Aggravating conditions***	A. No aggravating conditions	1
		B. Aggravating conditions that have a small effect on emissions	1.5		B. Aggravating conditions that have a small effect on emissions	1.5
		C. Aggravating conditions that have a large effect on emissions	2		C. Aggravating conditions that have a large effect on emissions	2

Product developers are provided with predefined options to describe and score each of the mentioned variables (Tables 2 and 3). Each of these options is assigned a weight that determines its contribution to the overall concern score ($\:{h}_{C,\:i\:}$ and $\:{e}_{C,\:i\:}$). The weighting of the variables and subsequent options is based on existing guidance and expert judgment. For example, following the ECHA Guidance [40] threshold values, a substance with the hazard category *Very persistent and very bioaccumulative (vPvB)* is assigned a higher weight than the category *Persistent and bioaccumulative.* All variables and their predefined options, along with their respective scores, are presented in Tables 2 and 3. The motivation for the selected variables and the weighing of their options is explained in full in Supplementary Material B.

The variables and options for describing and scoring them were developed to support product developers in describing the specific situation that applies to the product they are analysing. The purpose is to draw attention to factors that contribute to the concern score and can be influenced through design.

As users apply the tool during the early stages of product development, a relatively high level of uncertainty is inherent. To address this, an Uncertainty Score ($\:{h}_{U,\:i\:}$ for human health scenarios or $\:{e}_{U,\:i\:}$ for ecosystem health scenarios) (highlighted in blue in Fig. 5) is calculated for each exposure scenario, complementing its Concern Score ($\:{h}_{C,\:i\:}$ and $\:{e}_{C,\:i\:}$). This helps indicate data gaps while allowing flexibility when data is limited, inconclusive, or unavailable. When assessing each exposure scenario and selecting from the variable options (Tables 2 and 3), the product developer can also indicate the level of uncertainty for each variable. Each indication of uncertainty has a predefined weight (in points), as shown in Table 4 below. For example, if the variable $\:{e}_{{V}_{6,i}}$ *Frequency of exposure* is marked as chronic, but the product developer is not entirely sure the data point is correct, then its uncertainty weight can be marked as $\:{U}_{{e}_{{V}_{6,i}}}$= 2 points (medium uncertainty).

**Table 4:** Weights assigned to indicate the level of uncertainty for each variable (e.g., $\:{h}_{{V}_{1,i}}$, $\:{e}_{{V}_{1,i}}$, etc.). These weights are used to calculate the overall uncertainty score for each exposure scenario

Level of uncertainty	Description	Weight (pts)
No uncertainty	The data point is certain (within 10%)	1
Low uncertainty	The data point is a reasonable estimation based on other available data (10–50%)	1.5
Medium uncertainty	The data point is not a reliable estimation or comes from an unreliable source, and requires further research (50–200%)	2
High uncertainty	The data point is a guess to illustrate a scenario but requires further research (> 200%)	3

To calculate the uncertainty scores ($\:{h}_{U,\:i}$ and $\:{e}_{U,\:i}$) for the analysed exposure scenario, the tool multiplies the uncertainty levels (Table 4) indicated for each variable (Tables 2 and 3):

3 $$\begin{array}{c}{h}_{U,\:i\:}=\:{U}_{{h}_{{V}_{1,i}\:}}\:\bullet\:\:{U}_{{h}_{{V}_{2,i}\:}}\:\bullet\:\:{U}_{{h}_{{V}_{3,i}\:}}\:\bullet\:\:{U}_{{h}_{{V}_{4,i}\:}}\:\bullet\:\:{U}_{{h}_{{V}_{5,i}\:}}\:\bullet\:\:{U}_{{h}_{{V}_{6,i}\:}}\:\bullet\:\:{U}_{{h}_{{V}_{7,i}\:}}\:\bullet\:\:{U}_{{h}_{{V}_{8,i}\:}}\:\bullet\:\:{U}_{{h}_{{V}_{9,i}\:}}\end{array}$$

And

4 $$\begin{array}{c}{e}_{U,\:i\:}=\:{U}_{{e}_{{V}_{1,i}\:}}\:\bullet\:\:{U}_{{e}_{{V}_{2,i}\:}}\:\bullet\:\:{U}_{{e}_{\begin{array}{c}{V}_{3,i}\\\:\:\end{array}}}\:\bullet\:\:{U}_{{e}_{{V}_{4,i}\:}}\:\bullet\:\:{U}_{{e}_{{V}_{5,i}\:}}\:\bullet\:\:{U}_{{e}_{{V}_{6,i}\:}}\:\bullet\:\:{U}_{{e}_{{V}_{7,i}\:}}\:\bullet\:\:{U}_{{e}_{{V}_{8,i}\:}}\end{array}$$

A higher uncertainty score indicates that the concern score is inconclusive and requires further research, either by gathering additional data for the specific scenario or by identifying potential hotspots to be addressed through preventive design strategies. Product developers receive an interpretation guide to support the simultaneous assessment of concern and uncertainty scores. This interpretation guide is included in the MAPSSS *Tool protocol and steps*, available as Supplementary Material D.

##### Visualising and interpreting concern and uncertainty scores

For each exposure scenario, a concern score ($h_{c,\;i}$ and $e_{c,\;i}$) and an uncertainty score ($h_{U,\;i}$ and $e_{U,\;i}$) are calculated, reflecting the potential risk level and the confidence in the underlying data. As numerous scenarios may be assessed and the individual scores are not intended for quantitative interpretation, the tool consolidates the results into a three-tier scale (low, medium, high), indicating the overall level of concern. This supports the identification of risk hotspots, data gaps, and comparisons between reference products, alternatives, or new designs. The results can be visualised alongside each exposure scenario, as shown in Fig. 4. The concern scores are represented by small, medium, or large filled pink triangles, and uncertainty scores by open blue triangles of corresponding sizes (Fig. 3).

Product developers can use the following guidelines to interpret the maps and identify next steps:

- Prioritise high-scoring scenarios and assess their relevance to the product design; some may fall under the responsibility of other stakeholders, such as suppliers.
- Analyse score origins by reviewing the selected options to describe variables. If high scores result from aggravating conditions, preventing these may be a suitable strategy.
- Interpret combinations of concern and uncertainty scores:
  - Low concern – low uncertainty – The information is sufficient, and the scenario can be considered of low concern.
  - High concern – low uncertainty – The scenario represents a clear hotspot that requires mitigation through alternatives.
  - High concern – high uncertainty – Additional information is needed to confirm the concern; preventive measures should be considered.
  - Low concern – high uncertainty – The scenario demands caution. The low concern can be misleading and a result of a lack of data or an underestimation of risks. Therefore, additional information is likely necessary, and preventive measures should be considered.

#### Rules to Identify Environmental Impact Trade-Offs

The tool provides the means to perform a qualitative analysis to identify potential environmental trade-offs from design changes or mitigation strategies addressing the SoC. This allows product developers to reflect on the balance between safety and sustainability and to compare system maps of the reference product with those of alternatives or new designs. The analysis is guided by a set of rules based on LCA theory and literature, considering climate change and resource use as impact categories. The product developer is provided with an initial list of trade-off rules (Table 5) to analyse and identify any potential trade-offs on the system maps of each alternative. The assessment can draw on sources such as bills of materials, basic (open source) LCI databases, analysis of user behaviour, or product performance tests. Trade-offs are indicated on the maps by highlighting the relevant unit processes in green (Fig. 2).

**Table 5:** An initial list of rules to identify potential environmental impact trade-offs that occur as a consequence of reducing risks related to SoC

Production	Transport	Use	EoL
The extraction of the substitute material or additive is more impactful [41] *(e.g.*,* stainless-steel container instead of plastic with additives that are SoC)*	**Transportation impact is increased due to increase in mass**,** variety of materials**,** supplier location**, etc. [27, 42] *(e.g.*,* longer distances as a consequence of substitution*,* substitute for PVC cables is denser and requires a thicker layer)*	**The durability is reduced necessitating earlier replacement** [27, 41] *(e.g. substituting plastics with materials like glass or metal resulted in higher overall environmental impacts*,* partly due to increased material fragility*,* substitute additives in PVC cables reduce durability)*	**New processing steps or infrastructure are necessary** [27, 43] *(e.g. reducing SoC impacts by increasing useful life in electronics through applying composite materials can require additional steps like cleaning*,* sorting*,* and material separation during EoL processing)*
Processing is more complex or requires more steps and/or is energy intensive [27, 42] *(e.g. Reduction of VOC content in chemicals complicates the processing of the chemical*,* higher production costs and changes in the production methods are a consequence of substitute blowing agents)*		**The alternative requires maintenance or repairs** [27, 44] **(***e.g. material efficiency in electronics vs. reliability and safety*,* increasing the useful life of products can require additional maintenance and repair)*	**Processing waste/resources is more energy intensive** [41] *(e.g. recycling aluminium is more impactful than microplastic generating PET)*
More material is necessary to meet a function, due to performance, or to prevent risk [27, 42] *(e.g. small amounts of a toxic material vs. larger amount of a less toxic material*,* additional components to prevent risk)*		**The alternative has higher energy consumption / reduced efficiency / more waste generated** [27, 45, 46] *(e.g.*. *substitute refrigerant gases can have consequences on the appliance performance and energy consumption)*	**Processing techniques are not available (close by)** [47] *(e.g. disassembly of mattresses needs large ovens to melt adhesive which are only available in specific areas*,* additional logistical challenges)*
‘Known’ less sustainable material vs. ‘unknown’ potentially more sustainable material [27, 42] *(e.g.*,* Substitution of PFAS with non-fluorinated substances*,* often with an unknown safety profile)*			**The new material/product/chemical hinders circularity** [27, 48] *(e.g. Containment of components for safety inhibits disassembly*,* material is not recyclable)*
New impacts when transitioning to a service/digital product [27, 49] *(e.g.*,* additional infrastructure is necessary to meet the function)*			

#### Guidelines for Comparing Resulting Maps

The comparison of the maps can be performed once the reference product and one or more alternatives or new designs have been mapped. The comparison consists of a simultaneous analysis of the maps considering three aspects: (1) SoC safety, (2) risk trade-offs (considering uncertainty), and (3) potential environmental trade-offs, as defined by the tool development criteria in section 2.1.

Product developers can follow these guidelines for analysis:

- Observe if any changes to “contaminated” inputs and outputs are present. Especial attention should be given to inputs and outputs marked with blue (outline) rhomboids, indicating substances with unknown or inconclusive safety profiles. Marked outputs that exit the system boundary or lead to circular scenarios should be treated with caution, as they can create uncontrolled situations and unforeseen risks.
- Compare all resulting concern and uncertainty scores. Numerical results from the concern score calculator should not be used quantitatively, as this is a semi-quantitative assessment. The focus should be on reducing or eliminating risk hotspots while preventing uncertainty hotspots. Any new risk scenarios in alternative maps should also be evaluated.
- Analyse the potential environmental trade-offs among the alternatives. This streamlined form of LCA provides indicators to obtain further information and help prevent unforeseen impacts.

### Integration of the Tool Into the Product Development Process

**Fig. 6:**
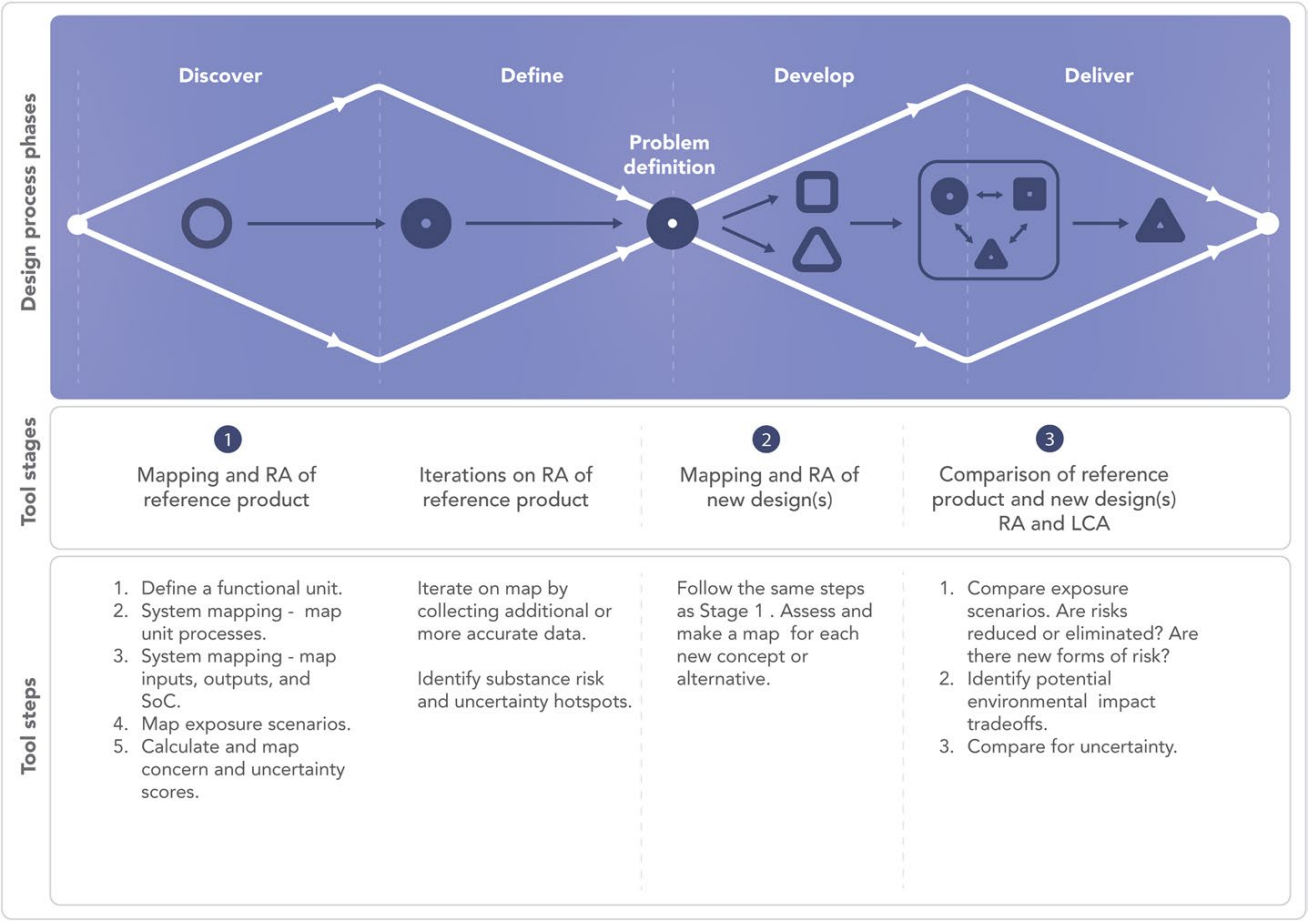
Stages, steps, and integration of the MAPSSS tool into the design process. The level of knowledge of the analysed product is represented by the circle filling in as the process progresses. The other geometrical figures represent new designs or alternatives

The *MAPSSS tool* is targeted at product developers to support them in dealing with the risks generated by SoC in products. It is an assessment tool that aims to provide indicators that highlight risk hotspots and support decision-making, especially in the early stages of the development process with high uncertainty. Based on this assessment, product developers can begin designing using the three strategies introduced earlier: avoid, reduce, and control [27]. Figure 6 provides an overview of the three main stages proposed for product developers to integrate the tool elements into the design process. Each of the tool stages contains a set of steps that correspond to the main goal or activity of the design process phase to which it belongs. A protocol document was also developed to guide product developers through these steps, which is available in Supplementary material D.

Prior to the use of MAPSSS, designers should define the goal and scope of the study, similar to established LCA protocols (e.g., Guinée et al. [32]). In this stage, the designer can define, for instance, the geographic and temporal coverage of the analysis, as well as the goal, such as the identification of risk hotspots in an existing product, the identification of specific knowledge gaps, or the comparison of alternatives. When developing the system map, the designer can also establish which unit processes are to be analysed and which fall out of the scope of the analysis.

- *Stage 1 - Mapping and Risk Assessment of the reference product*

During this stage, product developers map and assess the SoC risks for the reference product or design, using the mapping elements, concern and uncertainty score calculators, and interpretation guidelines. This process occurs during the Discover and Define phases, necessitating several iterations to gather all the necessary information for the assessment and establish a problem definition. It is assumed that the product developer is aware of or suspects the presence of a SoC in the reference product before commencing Stage 1. Stage 1 concludes with the identification of substance risks and uncertainty hotspots in the reference product.

- *Stage 2 - Mapping and Risk Assessment of new design(s)*

After developing concepts that address the risk hotspots identified during Stage 1, product developers can map and assess the associated substance risks during Stage 2. An assessment must be performed for each concept, following the same set of steps used to assess the reference product. During this stage, product developers also conduct a qualitative analysis to identify potential environmental trade-offs resulting from the design changes, applying a list of trade-off rules. This supports early identification of unintended environmental impacts. Stage 2 takes place during the Development phase of the design process.

- *Stage 3 - Comparison of maps of existing product and new design(s)*

After assessing the reference product and new concepts, product developers can proceed to compare the resulting maps. Stage 3 provides guidance for comparing risk mitigation, potential environmental trade-offs, and uncertainty. This stage occurs late in the Development phase of the design process and should provide product developers with information for comparing concepts and making informed decisions. Stage 3 should also provide uncertainty indicators that should be addressed throughout the Deliver phase of the process.

### Use of the Tool in an Example Case – Hospital Mattress Containing Flame Retardants

**Fig. 7:**
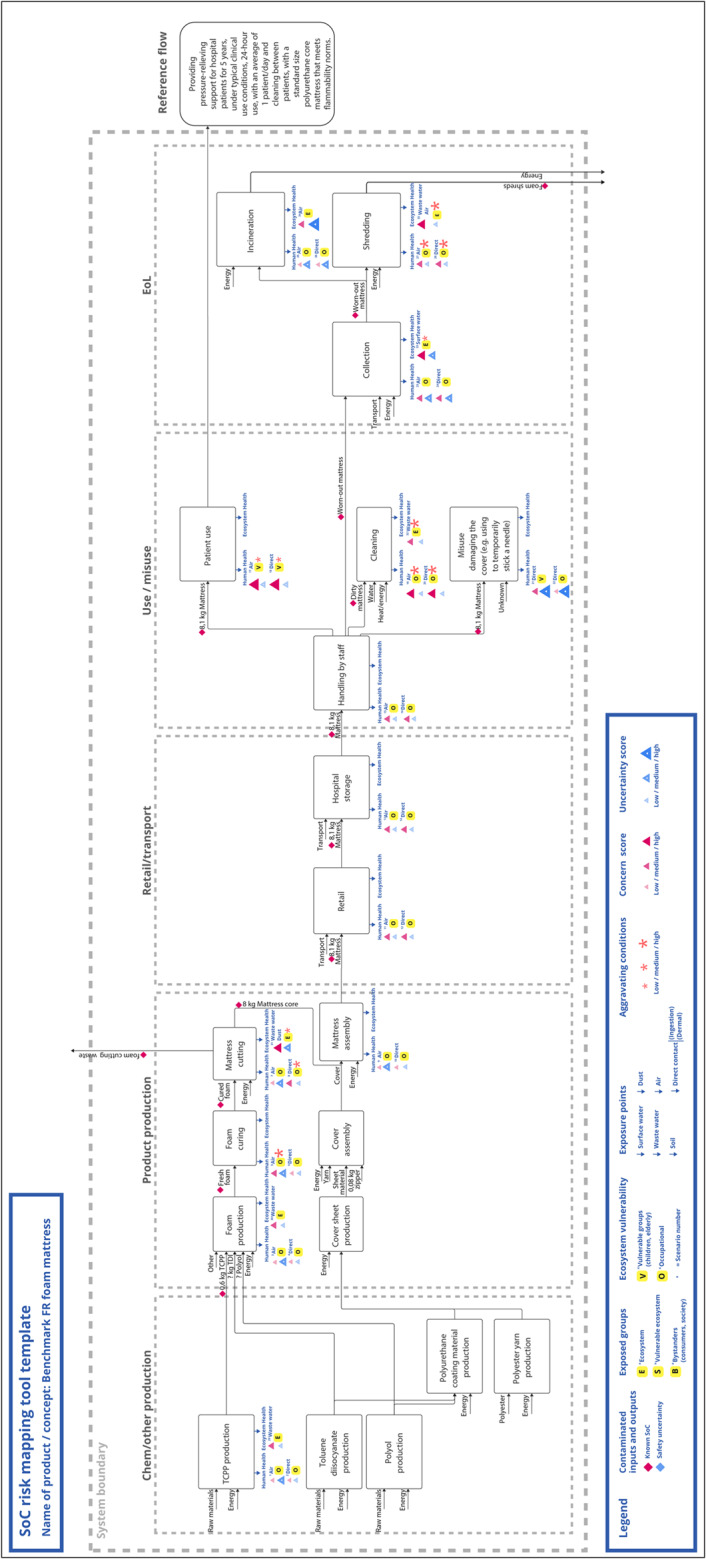
Resulting map of the reference hospital mattress containing TCPP as a flame retardant. The map illustrates the use of the tool elements, including system mapping of the product, indicators of contaminated inputs and outputs, exposure scenarios for human and environmental health, results for concern and uncertainty scores, and aggravating conditions

**Fig. 8:**
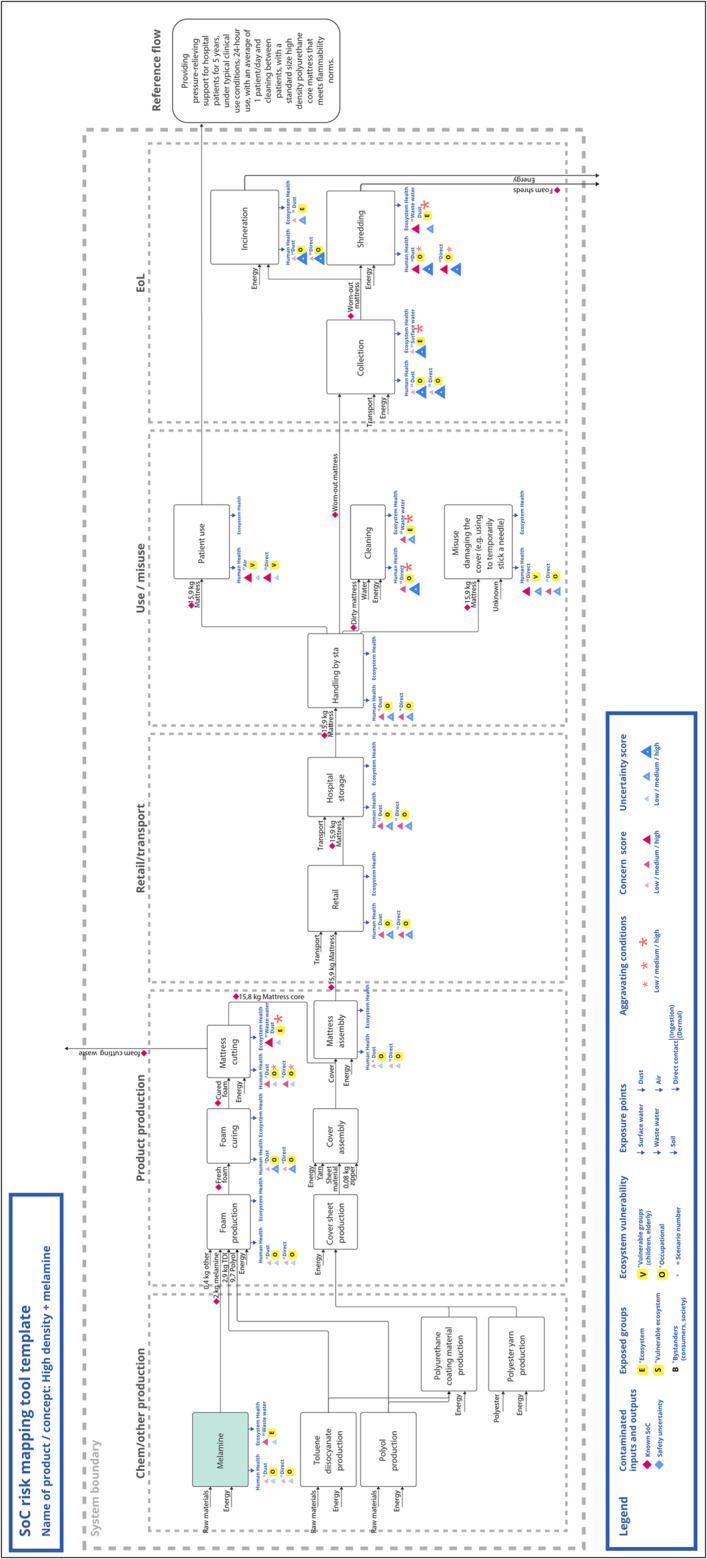
Resulting map of the alternative high-density foam hospital mattress containing melamine as a flame retardant. The map illustrates the use of the tool elements, including system mapping of the product, indicators of contaminated inputs and outputs, exposure scenarios for human and environmental health, results for concern and uncertainty scores, and aggravating conditions. Potential environmental trade-offs are indicated by unit processes highlighted in green

**Fig. 9:**
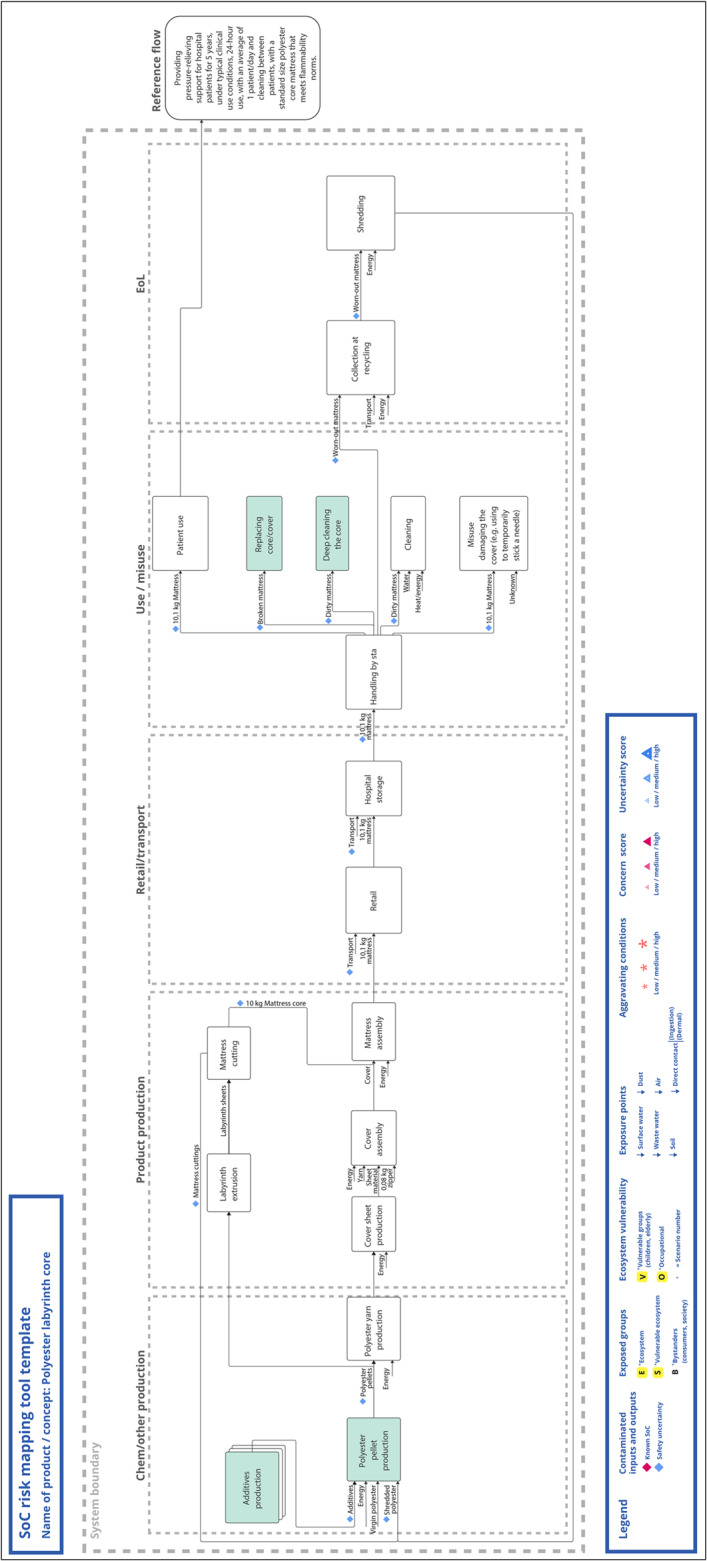
Resulting map of the alternative hospital mattress with a polyester labyrinth core. The map illustrates the use of the tool elements, including system mapping of the product, indicators of contaminated inputs and outputs, exposure scenarios for human and environmental health, results for concern and uncertainty scores, and aggravating conditions. Potential environmental trade-offs are indicated by unit processes highlighted in green

In this section, we present the application of the tool elements described above using a case example. While the case is used solely for illustrative purposes, the product and its alternatives represent real, commercially available examples. We present the resulting map of the reference product (Fig. 7), a polyurethane foam hospital mattress containing TCPP, a hazardous flame retardant, and the maps of two possible alternatives: a denser foam mattress with reduced flame-retardant content that uses melamine as a substitute for TCPP (Fig. 8), and a mattress with a polyester 3D structure that is inherently flame-retardant (Fig. 9). The functional unit defined to analyse the mattress case is: *Providing pressure-relieving support for hospital patients for 5 years*,* under typical clinical use conditions*,* 24-hour use*,* with an average of 1 patient/day and cleaning between patients*,* with a standard-size mattress that meets flammability norms.* Each map indicates the reference flow for each product. The data used are available in Supplementary Material A.

Figure 7 shows the processes, inputs, and outputs of the reference mattress system. In this specific case, TCPP contaminates several inputs and outputs, including some that extend beyond the system boundary, such as shredded foam from recycling and foam waste from cutting operations. Potential human health concerns arise, e.g., for vulnerable individuals in direct contact during use, for workers during curing and shredding, and for hospital staff during cleaning. Curing and cleaning could aggravate TCPP emissions, as heat can increase the substance’s volatility. The presence of water problematizes its solubility and consequent emissions. Environmental health concerns are highest for foam cutting and EoL processes, where the SoC is released into the air and wastewater. High uncertainty exists during misuse (cover damage) and EoL scenarios due to limited data on emissions and exposure.

Comparing all three maps (the TCPP reference mattress and two alternatives) reveals several points. Substituting TCPP with melamine offers little to no improvement, as melamine is also a SoC, contaminating multiple inputs and outputs and generating similar concerning scenarios, particularly when contaminated outputs cross the system boundary (Fig. 8). A slight improvement is observed during foam curing, as melamine is not as volatile. However, no improvements are seen in scenarios concerning patients during the use phase. In contrast, the polyester labyrinth alternative shows no concern scenarios. However, its inputs and outputs are flagged as uncertain because the safety data for some substances (such as water-repellent treatments) is inconclusive. Figure 9 also indicates that while the polyester structure reduces SoC risks, potential environmental trade-offs must be considered due to the change in core material and the need for additional cleaning and maintenance processes. Furthermore, Fig. 9 highlights a potential circular scenario where shredded foam re-enters the system, for which risks from recycled foam sources and other contaminants should be considered.

## Discussion

This study set out to develop a tool that integrates the principles of risk assessment (RA) and life cycle assessment (LCA) within a product development context. The following discussion examines how the resulting tool meets the predefined criteria established to achieve this aim, its contribution to the realm of Safe and Sustainable by Design (SSbD), and the study’s limitations and future research directions.

### Criteria Compliance

Here, we discuss how and to what extent the MAPSSS tool meets the criteria (C1-C6) established in section 2.1. The tool’s elements enable product developers to identify and visualise the risks posed by hazardous chemicals and materials across the entire product life cycle, including circular scenarios. Through the *concern score*, product developers can prioritise and locate risk hotspots (C1). There are situations where prioritising hotspots may still fail. For example, if the maps cannot be refined—because data on the SoC is missing or the value chain lacks transparency—uncertainty remains high across all exposure scenarios, making it impossible to identify hotspots. The tool may also fail this criterion when all exposure scenarios receive a high *concern score*. In this case, no hotspots can be identified, and thus no targeted solutions can be designed. This result, however, could indicate that the SoC is unacceptable for use altogether, suggesting that it should be avoided in the product developer’s or company’s full portfolio and that internal restricted substances lists should be established.

The tool captures uncertainties, including assumptions about processes or data points that are unavailable. The *uncertainty score* estimates their magnitude, and the map highlights data gaps and areas requiring further research (C2). Uncertainty is never considered negligible, especially since the tool itself is already a simplification of the much more detailed RA and LCA methods. Instead, the tool highlights high uncertainty as a risk. This aligns with other proposed approaches to uncertainty in SSbD, where uncertain scenarios are minimised as much as possible and highlighted as sensitive if uncertainty cannot be reduced [50]. The maps provide a visual overview of the entire life cycle(s) and the identified uncertainties attached to each exposure scenario, supporting both the analysis of the reference product and its potential alternatives (C3). Although the uncertainty is self-reported, which may potentially introduce under- or overestimation of the level of uncertainty, product developers are encouraged to assume the worst-case scenario. The *uncertainty score* can only decrease in an iteration of the map after new data is found. Making the *uncertainty score* an integral part of the tool aims to address some of the known challenges in uncertainty analysis and consequent poor application in LCA [51, 52].

Product developers can visually compare alternatives in terms of safety and potential environmental impact trade-offs using the lifecycle maps (C4). The tool thus enables transparency in the choices made by the product developer, supporting the assessment and comparison of interventions at different levels, such as alternative chemicals or materials, or alterations to the product design. The tool locates SoC and allows for prioritisation, which, to date, remains underrepresented in product development compared with environmental impact assessments based on, for instance, existing fast-track LCA methods such as the Idemat or Eco Audit Tools [53, 54]. This aligns with the SSbD approach in the JRC framework, which prioritises substance safety across the life cycle before addressing other environmental, social, or economic aspects [55].

Guidance is provided for when and how to apply the tool throughout the development process, considering an iterative approach (C5). Specific tool steps support specific phases of the product development process. The map facilitates an incremental, iterative approach to the assessment. Early analyses can be conducted even with limited data, drawing on assumptions, analogous cases, or qualitative observations. Naturally, this approach is not without risks. Product developers risk placing undue confidence in preliminary assessments and neglecting subsequent re-evaluations, having prematurely regarded product safety as resolved. Iteratively, these assessments need to be refined as more information becomes available. Oversimplification or the use of unrepresentative analogues are pitfalls, too. However, this is not unique to this tool; it is a known pitfall for lifecycle assessment in general [56]. Guidance for using the tool will outline critical and iterative thinking.

Finally, the tool supports a simplified scenario-based risk screening assessment, encouraging product developers to consider different stakeholders and contextual dimensions in their evaluations (C6). It inspires problem framing through hotspot identification and, subsequently, creative ideation for intervention options.

### Building on SSbD

This paper offers a product development perspective to complement existing approaches to SoC. The JRC SSbD framework takes safety and sustainability assessment as the starting point [55]. The *by-design* element in this framework refers mainly to the design of chemicals and materials, and the main target audience is industry experts in these processes. The product perspective is assumed only at specific levels of assessment, where it is a *necessary evil* rather than a perspective leveraged to enable safety and sustainability. In other words, the perspective introduced in the current paper enables a new set of experts to reflect on the topic at hand and positions product system design as an additional solution space beyond safer chemicals and materials, such as redesigning the product infrastructure or a circular business model [27].

Our approach begins with a reference product containing a SoC and aims to improve from a product perspective. This shifts the focus to the actual context in which a substance is used and encourages product developers to identify risks directly relevant to their product system. By doing so, opportunities for mitigation can be identified at the product level, which reduces reliance on the availability of safer chemicals. By treating uncertainty as a potential risk in our tool, we encourage product developers to adopt a precautionary mindset and consider combinations of mitigation strategies.

It is essential to acknowledge the distinctions in scope and assessment between the SSbD concept and the MAPSSS Tool. The JRC SSbD framework provides a much more comprehensive environmental sustainability assessment than our tool. It covers a broad set of impact categories, whereas our current focus is high-level and limited to rules of thumb for climate change and resource use. This is because our tool is designed to enable product developers to perform semi-quantitative assessments without expert involvement in early development. We realise that the simplification of RA and LCA in our tool may lead to biased designs in practice. The only way to avoid this is to work with interdisciplinary teams, including product developers, RA and LCA experts, and other relevant experts, as needed. In such situations, our tool can be used alongside assessments such as performance and other sustainability impacts. That, however, requires a fundamental shift in current business concepts and organization. Such development processes require a variety of resources that are currently unavailable.

While the JRC framework provides valuable guidance for SSbD implementation [1, 2], our tool could offer an entry point for product developers to contribute to the mitigation of SoC risks without requiring extensive expertise in LCA or RA. The MAPSSS tool supports early assessments that can be further developed with the involvement of RA and LCA experts, provided resources are available. This aligns with the JRC framework’s tiered approach. Our tool could provide a simplified and/or intermediate assessment step within its structure, depending on to-be-determined thresholds or criteria. For instance, in previously described situations of high uncertainty or high concern scores throughout the exposure scenarios, the product developer might escalate to the next assessment tier. In this way, the tool complements the existing framework. Considering the SSbD state-of-the-art overview by Apel et al. [3], MAPSSS aligns most closely with design stage screening tools that support iterative decision making during early and mid-innovation stages, rather than tools used for final assessment or verification. In this context, MAPSSS supports early identification of potential chemical risk hotspots and related trade-offs in situations where full RA and LCA are not yet available or feasible.

As stated in the introduction, SSbD requires multidisciplinary teams for its implementation, and MAPSSS could support the integration of product developers into these efforts. MAPSSS is intended for product developers working in different contexts, including multidisciplinary teams, independent practice, and design agencies. In each of these contexts, the tool is expected to facilitate communication between design and other areas of expertise relevant to SSbD.

SSbD is also addressed in ESPR (Ecodesign for Sustainable Products Regulation), from which we can expect future product design requirements regarding the use of substances with risks to the environment or human health. Today, ESPR establishes that the use of substances that impair circularity should be restricted, in accordance with the Union’s existing chemical safety assessments. However, it does not yet provide further information for product developers to operationalise these concepts. MAPSSS considers how SoC behave during several product lifecycles, inviting a broader thinking of substances that have effects on the environment and health, especially in circular scenarios. Within such a structure, MAPSSS could support the identification of hazardous substances, even if these have not yet been classified as such by the Registration, Evaluation, Authorisation, and Restriction of Chemicals (REACH) or Restriction of Hazardous Substances (RoHS) directives, since their potential effects are considered from a holistic perspective.

### Limitations and Recommendations

This research presents an initial version of the MAPSSS tool based on illustrative cases, literature, and expert input on the application of the assessment elements from RA and LCA, which makes it entirely theoretical. Its further development requires application by product developers in real, unsolved cases. Such testing should focus on enhancing usability and integrating it into the product development process. Further development should also consider improving the comparison step, focusing on usability, simultaneous visualisation of results, and systematic comparison guidelines.

At present, the tool is intended to be usable without prior expertise in chemicals, RA, or LCA. Although MAPSSS provides interpretation guidelines and highlights uncertainty scenarios as concerning, there is still a risk of misinterpretation among users with limited experience in RA and LCA methods and concepts. This should be further evaluated in future development steps of MAPSSS in collaboration with product developers, to define the level of knowledge required for responsible use of the tool and to assess whether the current tool elements adequately support safe use across different user knowledge levels. The main focus should remain on preventing substance-related risks from being overlooked and ensuring that uncertainty is not falsely interpreted as safety.

The concern score, which plays a central role in hotspot identification and the comparison of alternatives, has not been validated against real-world exposure and hazard data or the outcomes of full risk assessments. Therefore, the concern score should not be interpreted as a proxy for actual risk but rather as a screening level indicator, as MAPSSS is intended to support early decision-making and discussions between product developers and LCA and RA experts. Future validation should involve comparative studies in which the risks and environmental impacts of a product and its alternatives are assessed using both the MAPSSS tool and full RA and LCA approaches in parallel. Such comparisons could provide valuable insights into the tool’s accuracy in identifying hotspots and trade-offs. Iterations of case studies will, in due time, allow refinements, including, for example, refinements to the methodologies for concern and uncertainty scores and to the weights assigned to variables in the calculations.

The current assessment of environmental trade-offs enabled by the tool is limited to climate change and resource use, relying on rules of thumb rather than comprehensive analysis. In addition to environmental trade-offs, we foresee that, in the coming decades, social and economic sustainability will gain greater interest and thus lead to greater data availability. This will be an opportunity for the current tool to include more diverse data and assessment methods.

## Conclusion

Broad adoption of SSbD requires tiered, resource-efficient assessments, tailored to specific professionals’ needs. Early analyses, which are based on limited data and can be refined as more information becomes available, are essential. Streamlining is therefore necessary to support product development teams that may not have the means to perform resource-intensive assessments. Our tool addresses this need through its accessible, user-friendly interface, specifically tailored for product developers. The *MAPSSS tool* supports product developers in addressing the presence of SoC in products by using LCA and RA principles. It is an assessment and visualisation tool that enables the identification and evaluation of SoC risks, potential trade-offs, uncertainty levels, and environmental impacts throughout the product life cycle. Product developers are guided to identify risk hotspots in the reference product, supporting the development of mitigation strategies. They can later compare the reference product with new designs, modifications, or alternatives, in terms of safety and environmental trade-offs. The tool’s visual and iterative nature aligns with product development practices.

This work brings a product development perspective to current Safe and Sustainable by Design approaches, which have primarily focused on identifying alternatives to safer chemicals and materials. With this tool, an initial step toward developing a product-specific assessment tool for early design stages is taken, providing a foundation for future work that can strengthen the role of product developers in SSbD.

## Supplementary Information

Below is the link to the electronic supplementary material.

Supplementary Material A (DOCX 2.65 MB)

Supplementary Material B (DOCX 888 KB)

Supplementary Material C (XLSM 71.6 KB)

Supplementary Material D (DOCX 1.71 MB)

## Data Availability

All relevant data and resources related to this research are available through the supplementary materials.
