# Supplementary material for: Mapping Uncertain Risks and Impacts of Substances of Concern in Product Life Cycles - An SSbD Tool for Product Developers
Source: Circ Econ Sustain. 2026 Aug 3;6(4):372. doi: 10.1007/s43615-026-01077-w (PMC13433538; doi:10.1007/s43615-026-01077-w)
Supplement: Supplementary file 1 [file 43615_2026_1077_MOESM1_ESM.docx]

**Supplementary Material A – Mapping of cases and alternatives- background data**

This supplementary material contains the background data and sources used to analyze the two cases introduced in the main paper using the Mapping Assessment for Product Substance Safety and Sustainability (MAPSSS) tool: the mattress with flame retardants and the mulch film.

Table of Contents

[The mattress case	2](#_Toc212642834)

[The mulch film case	8](#_Toc212642835)

[Unit processes	8](#_Toc212642836)

[References	13](#_Toc212642837)

# The mattress case

Data about 4 design alternatives of care mattresses was collected through interviews, (confidential) document analysis and (grey) literature search. The data are aggregated into Table A1. Unavailable data points are indicated by a dash.

Functional unit: *Providing pressure-relieving support for hospital patients for 5 years, under typical clinical use conditions, 24-hour use, with an average of 1 patient/day and cleaning between patients, with a standard size mattress that meets flammability norms.*

The resulting lifecycle maps that include the assessment results can be seen in Figure A1a-c.

| **Mattress** | **Dimensions [mm]** | **Substances in core material** | **Core material** | **Core density**  **[kg/m^3^]** | **Core mass [kg]** | **Flame retardant** | **Flame retardant %** | **Cover material** | **Cover mass [kg]** | **Zipper mass** | **Yarn mass** | **Total mass [kg]** | **Notes on emissions and exposure** | **Notes on the lifecycle** |
| --- | --- | --- | --- | --- | --- | --- | --- | --- | --- | --- | --- | --- | --- | --- |
| 0. Benchmark PU + TCPP | Standard dimensions of:  2000 x 900 x 150 =  270*10^6^ mm^3^ | - Toluene diisocyanate (second largest component) - Polyols (majority) - Water - Catalyst mixture - Cross linkers mixture - (Assumed to be similar to higher density foam) | Polyurethane foam | 30 kg/m^3^  Density based on analogues. We know the density is lower than the PU foam case with decreased amount of FR (case 1). | 8 | Tris(2-chloroisopropyl) (TCPP) (RIVM, 2022) | According to Blum et al., (2019) the maximum amount found in PU foam is 7 % of the mass.  That equals 0,6 kg per mattress. | Polyester woven fabric with a polyurethane cover for waterproofing. Sewing thread is PP (Kerr et al., 2024) | 0,9 | 0,08 | 0,002 | 8,9 | - Exposure to TCPP is likely to occur through inhalation and via dermal exposure during the manufacturing or use (National Toxicology Program, 2023). - Exposures can occur in offices, homes, and other indoor environments from using consumer products (National Toxicology Program, 2023). - Children are considered more susceptible to ingestion because of increased object-to-mouth behaviors (National Toxicology Program, 2023). - This substance is harmful if swallowed, is harmful to aquatic life with long lasting effects, is suspected of causing cancer and is suspected of damaging fertility or the unborn child. Suspected to be persistent (European Chemicals agency, n.d.). | Uncertain what happens at end of life, as some sources say it is not desirable to incinerate FR foam, nor is it safe to recycle the FR foam because this can be applied in e.g. children’s playgrounds. Landfill is banned in NL.  Uncertain how much of the SoC is kept inside the mattress by the cover. |
| 1. High density PU + melamine |  | - Toluene diisocyanate - Polyols - Water - Catalyst mixture - Cross linkers mixture - Silicon stabilizers - Melamine (12,89%) | Polyurethane foam | 55 kg/m^3^ | 15 | Melamine | 12.89%, which equals to about 2 kg | Polyester woven fabric with a polyurethane cover for waterproofing. Sewing thread is PP (Kerr et al., 2024) | 0,9 | 0,08 | 0,002 | 15,9 | - Melamine is a substance of very high concern (SVHC) - This substance is suspected of damaging fertility or the unborn child. - Suspected to be Carcinogenic - Under assessment as Persistent, Bio accumulative and Toxic (assumed in this assessment to be true with medium uncertainty) - Under assessment as Endocrine Disrupting (*Melamine*, n.d.) - May be considered as non-persistent or very persistent with half-life ranging from 46 to 211 days at 20 °C depending on the soil type - Potential groundwater contaminant, due to a low log octanol carbon coefficient (Koc) (Lütjens et al., 2023). - Solubility in water is depending on temperature. - The estimated daily intakes of these chemicals via dust ingestion for occupational e-waste recycling workers were 2.5–9 times higher than the estimated daily intakes for adult residents (Li et al., 2022). | Uncertain what happens at end of life, as some sources say it is not desirable to incinerate FR foam, nor is it safe to recycle the FR foam because this can be applied in e.g. children’s playgrounds. Landfill is banned in NL.  Uncertain how much of the SoC is kept inside the mattress by the cover |
| 2. Recycled PU |  | - Glue - Foam shreds | Rebound polyurethane foam + prepolymer adhesive (isocyanate-polyol reaction product) (Duffy & Stout, 1976) | Around 50 kg/m^3^ | 13,5 | Unknown which additives are present in the rebounded foam, the recycling is open loop. Adheres to MDR biocompatibility standards. | Unknown | Polyester woven fabric with a polyurethane cover for waterproofing. Sewing thread is PP (Kerr et al., 2024) | 0,9 | 0,08 | 0,002 | 14,4 | - High uncertainty, as there is no known FR *added,* but foam could be sourced anywhere - Worst case is assumed: unintended FR are present, where most common FR are assumed (TCPP) - The mattresses go through the same high temperatures - Potentially more emissions can happen due to the different quality of the rebounded foam | Open loop recycling.  Less known about durability of the rebound foam. |
| 3. Polyester |  | - Polyester elastomer - Finish solution | Polyester | 37 kg/m^3^ | 10 | No added FR. Other SoC are unknown. Mattresses that use this core have received OEKO-TEX certification. | None | Polyester mono material | 0,2 | 0,07 | - | 10,3 | - There are no FR added, but because there is no foam used, major uncertainty is introduced - No data about potential emissions available on this specific material - Microplastics (inhalation and digestion main exposure routes) could be generated | Closed loop recycling.  Washing of the core and replacement of the cover is introduced. |

Table A1. Data used for the reference case mattress and three alternatives

*Figure A1a. Resulting map of the reference hospital mattress containing TCPP as a flame retardant. The map illustrates the use of the tool elements, including system mapping of the product, indicators of contaminated inputs and outputs, exposure scenarios for human and environmental health, results for concern and uncertainty scores, and aggravating conditions.*

*Figure A1b. Resulting map of the alternative hospital mattress with a rebound foam core. The map illustrates the use of the tool elements, including system mapping of the product, indicators of contaminated inputs and outputs, exposure scenarios for human and environmental health, results for concern and uncertainty scores, and aggravating conditions. Potential environmental trade-offs are indicated by unit processes highlighted in green.*

*Figure A1c. Resulting map of the alternative hospital mattress with a polyester labyrinth core. The map illustrates the use of the tool elements, including system mapping of the product, indicators of contaminated inputs and outputs, exposure scenarios for human and environmental health, results for concern and uncertainty scores, and aggravating conditions. Potential environmental trade-offs are indicated by unit processes highlighted in green.*

*Figure A1d. Resulting map of the alternative high-density foam hospital mattress containing melamine as a flame retardant. The map illustrates the use of the tool elements, including system mapping of the product, indicators of contaminated inputs and outputs, exposure scenarios for human and environmental health, results for concern and uncertainty scores, and aggravating conditions. Potential environmental trade-offs are indicated by unit processes highlighted in green.*

# The mulch film case

Here, the data used to assess the mulch film case is presented.

Baseline and alternatives to be considered in the analysis are presented in Table A2.

*Table A2. The physical characteristics of the reference product of PE mulch film and of its alternatives.*

|  | Scenario (Dada et al., 2025; Dong et al., 2022; Tan et al., 2023) | Thickness (Dong et al., 2022; Tan et al., 2023) | Density (Tan et al., 2023) | Weight (needed for FU) | Origin (Serrano-Ruiz et al., 2021) | EoL (Dong et al., 2022) |
| --- | --- | --- | --- | --- | --- | --- |
| 1 | PE (most common non-biodegradable) | 0.01 mm | 0.92 g/cm3 | 73.6kg | Fossil | Nature |
| 2 | PBAT (most common biodegradable) | 0.012 mm | 1.26 g/cm3 | 120.9kg | Fossil | Nature |
| 3 | PE (Plus recovery system) | 0.01 mm | 0.92 g/cm3 | 73.6kg | Fossil | Landfill  Recycling |

Functional unit:

1 ha (10,000 m2) of cropland of maize per year (1 planting cycle per year) – 8000 m2 of X mulch film

80% of maize cropland is covered = 8000 m2

- Conditions need to be defined in the use stage. Considering mechanical degradation, photo degradation, and other environmental conditions such as temperature, soil, etc.
- Conditions differ from one region to the other, highlighting the importance of mapping with LOCAL information as much as possible (Convertino et al., 2024; de Sadeleer & Woodhouse, 2024).

## Unit processes

Table A3 presents the process involved in the production of the reference mulch film and its alternatives.

*Table A3. The unit processes involved in the production of the reference product of PE mulch film and of its alternatives.*

|  | Scenario (Dada et al., 2025; Dong et al., 2022; Tan et al., 2023) | Mass | Polymer production | Mulch film production | Use of mulch film | EoU in Nature | EoU  Collection | EoL  Recycling |
| --- | --- | --- | --- | --- | --- | --- | --- | --- |
| 1 | PE (most common non-biodegradable) | 73.6kg | X | x | x | x |  |  |
| 2 | PBAT (most common biodegradable)  No data in ecoinvent – it is a polyester so I used that data for exchanges. | 120.9kg | x | x | x | x |  |  |
| 3 | PE (Plus recovery system) | 73.6kg | x | x | x |  | x | X 18.2% residue  Impurity content 80% moisture content 5.3% (Dong et al., 2022) |

PE granulate <https://ecoquery.ecoinvent.org/3.11/cutoff/dataset/5573/exchanges>

PE-recycling <https://ecoquery.ecoinvent.org/3.11/cutoff/dataset/92667/exchanges>

Film production <https://ecoquery.ecoinvent.org/3.11/cutoff/dataset/5196/exchanges>

Polyester resin <https://ecoquery.ecoinvent.org/3.11/cutoff/dataset/5544/documentation>

PBAT production (BDO, AA, and PTA) (Luo et al., 2024).

The resulting lifecycle maps that include the assessment results can be seen in Figures A2a-c.

*Figure A2a. Resulting map of the reference mulch film. The map illustrates the use of the tool elements, including system mapping of the product, indicators of contaminated inputs and outputs, exposure scenarios for human and environmental health, results for concern and uncertainty scores, and aggravating conditions.*

*Figure A2b. Resulting map of the alternative mulch film where the film is collected at end of life. The map illustrates the use of the tool elements, including system mapping of the product, indicators of contaminated inputs and outputs, exposure scenarios for human and environmental health, results for concern and uncertainty scores, and aggravating conditions. Potential environmental trade-offs are indicated by unit processes highlighted in green.*

*Figure A2c. Resulting map of the alternative mulch film where the film is made of PBAT. The map illustrates the use of the tool elements, including system mapping of the product, indicators of contaminated inputs and outputs, exposure scenarios for human and environmental health, results for concern and uncertainty scores, and aggravating conditions. Potential environmental trade-offs are indicated by unit processes highlighted in green.*

# References

Blum, A., Behl, M., Birnbaum, L. S., Diamond, M. L., Phillips, A., Singla, V., Sipes, N. S., Stapleton, H. M., & Venier, M. (2019). Organophosphate Ester Flame Retardants: Are They a Regrettable Substitution for Polybrominated Diphenyl Ethers? In *Environmental Science and Technology Letters* (Vol. 6, Issue 11, pp. 638–649). American Chemical Society. https://doi.org/10.1021/acs.estlett.9b00582

Convertino, F., Carroccio, S. C., Cocca, M. C., Dattilo, S., Dell’Acqua, A. C., Gargiulo, L., Nizzetto, L., Riccobene, P. M., Schettini, E., Vox, G., Zannini, D., & Cerruti, P. (2024). The fate of post-use biodegradable PBAT-based mulch films buried in agricultural soil. *Science of The Total Environment*, *948*, 174697. https://doi.org/10.1016/J.SCITOTENV.2024.174697

Dada, O. I., Habarakada Liyanage, T. U., Chi, T., Yu, L., DeVetter, L. W., & Chen, S. (2025). Towards sustainable agroecosystems: A life cycle assessment review of soil-biodegradable and traditional plastic mulch films. *Environmental Science and Ecotechnology*, *24*, 100541. https://doi.org/10.1016/J.ESE.2025.100541

de Sadeleer, I., & Woodhouse, A. (2024). Environmental impact of biodegradable and non-biodegradable agricultural mulch film: A case study for Nordic conditions. *International Journal of Life Cycle Assessment*, *29*(2), 275–290. https://doi.org/10.1007/S11367-023-02253-Y/FIGURES/6

Dong, H., Yang, G., Zhang, Y., Yang, Y., Wang, D., & Zhou, C. (2022). Recycling, disposal, or biodegradable-alternative of polyethylene plastic film for agricultural mulching? A life cycle analysis of their environmental impacts. *Journal of Cleaner Production*, *380*, 134950. https://doi.org/10.1016/J.JCLEPRO.2022.134950

Duffy, R. D., & Stout, E. C. (1976). *POLYURETHANE PREPOLYMERS, REBONDED POLYURETHANE FOAMAND METHODS OF FORMING THE SAME RELATED APPLICATION*.

European Chemicals agency. (n.d.). *Tris(2-chloro-1-methylethyl) phosphate*. European Union Agency Website. Retrieved June 16, 2025, from https://echa.europa.eu/nl/substance-information/-/substanceinfo/100.033.766

Kerr, A., Meßner, L., Van Der Mars, S., Verstegen, W., Parra, M. F., Kuppen, M., & Agrawal, P. (2024). *Life Cycle Analysis: Functional Mattress for the Healthcare Sector for a Circular Economy Research Report Innovative Textile Development In Cooperation with the Sustainable & Functional Textiles Research Group Project 3*.

Li, J., Gao, X., He, Y., Wang, L., Wang, Y., & Zeng, L. (2022). Elevated emissions of melamine and its derivatives in the indoor environments of typical e-waste recycling facilities and adjacent communities and implications for human exposure. *Journal of Hazardous Materials*, *432*. https://doi.org/10.1016/j.jhazmat.2022.128652

Luo, C., Zhou, Y., Chen, Z., Bian, X., Chen, N., Li, J., Wu, Y., & Yang, Z. (2024). Comparative life cycle assessment of PBAT from fossil-based and second-generation generation bio-based feedstocks. *Science of The Total Environment*, *954*, 176421. https://doi.org/10.1016/J.SCITOTENV.2024.176421

Lütjens, L. H., Pawlowski, S., Silvani, M., Blumenstein, U., & Richter, I. (2023). Melamine in the environment: a critical review of available information. In *Environmental Sciences Europe* (Vol. 35, Issue 1). Springer. https://doi.org/10.1186/s12302-022-00707-y

*Melamine*. (n.d.). Retrieved June 16, 2025, from https://echa.europa.eu/sl/substance-information/-/substanceinfo/100.003.288

National Toxicology Program. (2023). *NTP Technical Report on the Toxicology and Carcinogenesis Studies of an Isomeric Mixture of Tris(chloropropyl) Phosphate Administered in Feed to Sprague Dawley (Hsd:Sprague Dawley® SD®) Rats and B6C3F1/N Mice*. https://doi.org/10.22427/NTP-TR-602

RIVM. (2022). *Een veilige en duurzame matras begint bij het ontwerp*. https://www.rivm.nl/sites/default/files/2022-07/RIVM-Brochure-Matrassen-V5-TG.pdf

Serrano-Ruiz, H., Martin-Closas, L., & Pelacho, A. M. (2021). Biodegradable plastic mulches: Impact on the agricultural biotic environment. *Science of The Total Environment*, *750*, 141228. https://doi.org/10.1016/J.SCITOTENV.2020.141228

Tan, Q., Yang, L., Wei, F., Chen, Y., & Li, J. (2023). Comparative life cycle assessment of polyethylene agricultural mulching film and alternative options including different end-of-life routes. *Renewable and Sustainable Energy Reviews*, *178*, 113239. https://doi.org/10.1016/J.RSER.2023.113239
