# Supplementary material for: Mapping Uncertain Risks and Impacts of Substances of Concern in Product Life Cycles - An SSbD Tool for Product Developers
Source: Circ Econ Sustain. 2026 Aug 3;6(4):372. doi: 10.1007/s43615-026-01077-w (PMC13433538; doi:10.1007/s43615-026-01077-w)
Supplement: Supplementary file 2 [file 43615_2026_1077_MOESM2_ESM.docx]

**Supplementary material B – Adaptation of risk assessment for product developers**

This supplementary material includes the detailed description of the rationale behind the adaptation of the RA framework for product developers and the development of the concern and uncertainty score calculators.

Table of Contents

[1.	Adaptation of the RA framework for product developer	2](#_Toc214877904)

[2.	Variables to calculate the concern score	6](#_Toc214877905)

[3.	Uncertainty score	9](#_Toc214877906)

[4.	References	11](#_Toc214877907)

## 1. Adaptation of the RA framework for product developer

Chemical risk assessment is not a static or one-size-fits-all process; rather, it is inherently adaptable, shaped by its intended purpose, the nature and availability of data, and practical constraints. As noted in established literature, risk assessments can range from large-scale national analyses of pollutants to highly localized evaluations of specific contamination events, and from retrospective analyses to forward-looking evaluations of emerging substances (Van Leeuwen & Vermeire, 2007). Inspired by this inherent flexibility, we developed a tool that adapts the original RA framework to meet the product developer’s needs (Bolaños Arriola et al., 2023), considering their workflows, scope of their capabilities, and resources available to them when assessing chemical-related risks. The tool, therefore, excludes quantitative forms of assessment that rely on complex inputs, or data obtained from lab and field tests.

Initially, the Mapping Assessment for Product Substance Safety and Sustainability (MAPSSS) tool provides a system for developers to identify and visually describe exposure scenarios throughout the product’s life cycle (Figure B1). Exposure scenarios are defined by three variables: whether humans and/or ecosystems are impacted, the points at which exposure can occur (e.g., soil, water), and the exposed population (e.g., occupational, bystanders, vulnerable ecosystems) (Figure B1). Developers can define multiple exposure scenarios per unit process as indicated in the example in Figure B2. For human health, a scenario is created for each exposed group of people; for ecosystem health, one scenario per unit process is considered. The example in Figure B2 shows three scenarios: two for human health (both via air, but affecting two different groups: bystanders and occupational) and one for ecosystem health (via water and soil). An additional specification (pink asterisk) can be added if aggravating conditions are suspected, e.g., temperature or humidity variation, wear and tear from behavior or machinery, or interaction with other substances during maintenance. Developers should assess whether design-specific conditions (e.g., use at high temperatures, shredding at end of life) combined with intrinsic substance characteristics (e.g., solubility, volatility, adsorption) may aggravate emissions. With these elements, the tool leverages the ability of product developers to consider a variety of product specific scenarios, including relevant stakeholders, and other aspects such as contextual factors, and behavior.

*Figure B1. Visual elements to map and describe exposure scenarios, indicating whether human health or environmental health is affected (scenario type), the exposed groups (exposed population or ecosystem vulnerability), exposure points, potential presence of aggravating conditions (e.g., heat, misuse, etc.), and resulting concern and uncertainty scores.*

Figure B2. *Proposed convention to apply the tool’s mapping elements. The example depicts a unit process with a contaminated input and output, and describes three exposure scenarios, two for human health via air to bystanders and occupational, and one for ecosystem health via water and soil. Aggravating conditions appear to be present for the occupational exposure scenario.*

Once the exposure scenarios have been defined by the developer, these can be further analyzed using what we call the concern score calculator. **The aim of the concern score is to assess the different exposure scenarios that occur throughout the lifecycle of the product, distinguishing between scenarios that impact human health and scenarios that impact ecosystem health. Through a semi-quantitative approach, developers can translate qualitative information on the characteristics and behavior of the substance throughout the life cycle of the product, into semi-quantitative scores. The scoring system is based on pre-defined variables that include options to describe and assess exposure scenarios through pre-assigned weights (Tables B1 and B2). The resulting scores can be used to help developers identify potential risk hotspots, and compare different risk scenarios within a design or its alternatives. These results can be further used to inform the development of design interventions that address risk.**

The concern score is a methodological adaptation of the original risk assessment framework. Figure B3 gives an overview of how elements of general risk assessment (RA) were adapted to be suitable for product developers. In this document we further describe the working principles of the concern score calculator, its variables, and the rationale behind the adaptation of the RA framework. Currently, the concern score calculator is made available to developers as an Excel template (Supplementary material C) which incorporates all elements and calculations described below.

*Figure B3. Overview of how the methodological RA framework (as described for example by* Boersema & Reijnders, (2009*) was modified for product developer use. The top panel depicts the RA framework, and the bottom panel depicts the modified approach to calculate the concern score including all considered variables to assess human health and ecosystem health related risks. Variables marked with a star have a weight in the concern score calculation, while those without it are only descriptive. The concern, effect, and exposure scenario scores are highlighted in red, while all uncertainty scores are highlighted in blue.*

**Hazard identification**

Hazard identification is meant to identify the adverse effects that a substance is inherently able to cause on humans and/or environment (Boersema & Reijnders, 2009; Van Leeuwen & Vermeire, 2007). In traditional RA, such is done through analysis of data about health effects (e.g. carcinogenicity, mortality) or environmental damage (e.g. population growth of a species fitness). Data are gathered in lab studies or from in situ sources (e.g. fish or air samples in offices), going as far as characterization of the behavior of a substance in specific tissues, and verified within the field in cases of high pollution hotspots and accidents. For the purpose of this tool, we can assume the substance of concern (SoC) and its hazard have been previously identified by means of bill of materials (BOM) analysis and available hazard data. This element of risk assessment is thus not explicitly supported by the tool.

**Risk Characterization**

Risk characterization in the original RA framework, is based on the exposure and effect assessment results (Figure 3). The result is often expressed as a risk quotient (e.g., PEC/PNEC for environmental risk or exposure/NEL for human health), which compares exposure levels to safe thresholds.

The concern score calculator follows two different pathways to support the assessment of scenarios that impact human health and scenarios that impact ecosystem health. In Figure B1, we see that a human health concern score ($h_{C, i}$) or an ecosystem health concern score ($e_{C, i}$), are equivalent to the risk characterization step in the RA framework. Here, *i* refers to a particular SoC. Concern scores should be calculated for each of the exposure scenarios previously defined by the developer in the map. A concern score ($h_{C, i}$ and $e_{C, i}$) is obtained by multiplying the results from the Effect score ($h_{A, i}$ and $e_{A, i}$) with the results obtained from the Exposure score ($h_{B, i}$ and $e_{B, i}$):

$\begin{aligned} h_{C, i}= h_{A, i} \cdot h_{B, i}\#\left( 1 \right) \end{aligned}$

or $\begin{aligned} e_{C, i}= e_{A, i} \cdot e_{B, i}\#\left( 2 \right) \end{aligned}$

**Effect Assessment**

The effect assessment step in risk assessment, which determines the relationship between exposure level and the incidence and severity of effects, typically results in a Predicted No Effect Concentration (PNEC) or Acceptable Daily Intake (ADI). Effect assessment relies on complex inputs, including quantitative structure-activity relationships (QSARs), in vitro tests, animal and field studies, or epidemiological data—each potentially producing different dose-response relationships for various toxic effects (Guinée et al., 2017; Van Leeuwen & Vermeire, 2007).

For product developers, it is relevant to identify the type and severity of the effects of the chemicals present in the analyzed product. To achieve this based on the information that is accessible to them, we have adapted this step of the original framework. The effect score ($h_{A, i}$ and $e_{A, i}$), which is equivalent to the effect assessment step in the RA framework, is calculated by multiplying three variables (Figure B3 and Table B1). In case of a human health scenario, the human health hazard category of the substance ($h_{{V_{1,i}}}$), its persistence/mobility/accumulation characteristics ($h_{{V_{2,i}}}$), and the exposed population ($h_{{V_{3,i}}}$) determine the effect score ($h_{A, i}$). In case ecosystem health is affected, the ecosystem health hazard category of the substance ($e_{{V_{1,i}}}$), its persistence/mobility/accumulation characteristics ($e_{{V_{2,i}}}$), and the ecosystem vulnerability ($e_{{V_{3,i}}}$) determine the effect score ($e_{A, i}$).

**Exposure Assessment**

Exposure in conventional RA (left panel, Figure B1) can be assessed by measuring or estimating exposure concentrations, based on measuring or estimating emission rates. These estimations incorporate theoretical or experimental data based on identified potential pathways a substance follows and its behavior (so-called *fate*), which results in a predicted dose to humans, or an environmental compartment. The target organ or organism then interacts with the exposure concentration that is bioavailable. Depending on the substance and the relevant exposure scenarios, this assessment involves describing the nature and size of the populations or compartments (e.g. soil, surface water) exposed, and the magnitude and duration of their exposure. These predictions are calculations often done with multimedia models and can be modified or extended depending on the available data and knowledge. Lack of data, however, shouldn’t prevent or limit actions of risk reduction. Qualitative estimations can be used in early stages of exposure analysis, be it with inherent larger uncertainties. For instance, the Swedish Chemical Agency advices to consider the following variables when analyzing exposure scenarios qualitatively (Swedish Chemical Agency, 2020):

- Volume of the substance produced or circulating society
- Use pattern
- Mapping of the uses of the chemical
- Use conditions
- Risk management measures at the site of use
- Exposure estimation/assessment of chemical pathways

Product developers are equipped to make qualitative observations to describe the behavior of the substance throughout the lifecycle of the product using the above variables. Furthermore, product developers have the information that is relevant to the specific application they are analyzing, considering involved stakeholders and contextual factors. The exposure scenario scoring process follows this approach.

The exposure score ($h_{B, i}$ and $e_{B, i}$), which is equivalent to the exposure assessment step in the RA framework, is calculated by multiplying several variables (Figure B3 and Table B2). In case of a human health scenario, the exposure score ($h_{B, i}$) considers four variables describing exposure ($h_{{V_{4,i}}}$ to $h_{{V_{7,i}}}$) and two variables describing emissions ($h_{{V_{8,i}}}$ and $h_{{V_{9,i}}}$) (Table B2). In case of an ecosystem health scenario, the exposure score ($e_{B, i}$) considers three variables describing exposure ($e_{{V_{4,i}}}$ to $e_{{V_{6,i}}}$), and two describing emissions ($e_{{V_{7,i}}}$ and $e_{{V_{8,i}}}$) (Table B2).

## 2. Variables to calculate the concern score

Product developers are provided with predefined options to describe and score each of the mentioned variables (Tables B1 and B2). Each of these options is assigned a weight that determines its contribution to the overall concern score ($h_{C, i}$ and $e_{C, i}$). The weighting of the variables and subsequent options is based on existing guidance and expert judgment. For example, following ECHA Guidance (European Chemicals Agency, 2014) threshold values, a substance with the hazard category *Very persistent and very bioaccumulating (vPvB)* is assigned a higher weight than the category *Persistent and bioaccumulative.* All variables and their predefined options with their respective scores are presented in Tables B1 and B2. The motivation for the selected variables and the weighing of their options is explained below.

The variables and the options to describe and score them, were developed with the intention of supporting developers in describing the specific situation that applies to the product they are analyzing. Additionally, the intention is to draw attention to factors that contribute to the concern score and can be influenced through design.

*Table B1. Variables to calculate the effect score (*$h_{A, i}$*or* $e_{A, i}$*) as indicated in Figure 5. Each variable has predefined options that the product developer can select from to describe and assess them. Each option has an assigned weight (points). For example, if the variable* $e_{{V_{1,i}}}$*Hazard category is marked as toxic to the aquatic environment, then* $e_{{V_{1,i}}}$*= 4pts.*

| **Human health Effect scoring (**$\boldsymbol{h}_{\boldsymbol{A, i}}$**),** $h_{A, i}=h_{V_{1,i}}\cdot h_{V_{2,i}}\cdot h_{{V_{3,i}}}$ | | | **Ecosystem health Effect scoring (**$\boldsymbol{e}_{\boldsymbol{A, i}}$**),** $e_{A, i}=e_{{V_{1,i}}}\cdot e_{V_{2,i}}\cdot e_{V_{3,i}}$ | | |
| --- | --- | --- | --- | --- | --- |
| ***Variable*** | **Options to describe variable** | ***Weight***  ***(pts)*** | **Variable** | **Options to describe variable** | **Weight (pts)** |
| ***(***$\boldsymbol{h}_{{\boldsymbol{V}_{\boldsymbol{1,i}}}}$***)***  ***Hazard category*** | A. Systemic health hazards: Carcinogenic, mutagenic or toxic to reproduction (CMR) | *4* | **(**$\boldsymbol{e}_{\boldsymbol{V}_{\boldsymbol{1,i}}}$***)***  ***Hazard category*** | A. Toxic to aquatic environment | 4 |
|  | B. Physical hazards (explosive, corrosive, flammable, gas under pressure, oxidizing) | 2 |  | B. Damage to the ozone layer | 2 |
|  | C. Direct health hazards: acute toxicity, specific organ toxicity, skin corrosion, skin or respiratory sensitization, eye damage | 2 |  |  |  |
| ***(***$\boldsymbol{h}_{{\boldsymbol{V}_{\boldsymbol{2,i}}}}$***) Persistence / mobility / bioacc.*** | A. Very persistent and very bioaccumulating or Very Persistent, very Mobile (vPvB or vPvM) | 4 | ***(***$\boldsymbol{e}_{\boldsymbol{V}_{\boldsymbol{2,i}}}$***) Persistence / mobility / bioacc.*** | A. Very persistent and very bioaccumulating or Very Persistent, very Mobile (vPvB or vPvM) | 4 |
|  | B. Persistent, bioaccumulating or mobile, and toxic (PBT or PMT) | 3 |  | B. Persistent, bioaccumulating or mobile, and toxic (PBT or PMT) | 3 |
|  | C. Not applicable | 1 |  | C. Not applicable | 1 |
| ***(***$\boldsymbol{h}_{{\boldsymbol{V}_{\boldsymbol{3,i}}}}$***)***  ***Exposed population*** | A. Vulnerable groups (children, pregnant, etc.) | *3* | ***(***$\boldsymbol{e}_{\boldsymbol{V}_{\boldsymbol{3,i}}}$***) Ecosystem vulnerability*** | A. Vulnerable ecosystem | 3 |
|  | B. People (consumers, society) | 1.5 |  | B. Ecosystem | 1.5 |
|  | C. Occupational | 1.5 |  |  |  |

*Table B2. Variables to calculate the exposure scenario score (*$h_{B, i}$ and $e_{B, i}$*) as indicated in Figure 5. Each variable has predefined options that the product developer can select from to describe and assess them. Each option has an assigned weight (points). For example, if the variable* $e_{V_{6,i}}$ *Frequency of exposure is marked as chronic, then* $e_{V_{6,i}}$ *= 2.5 points. Variable* $e_{V_{5, i}}$ *gets an accumulative weight of +1 for each selected exposure point (e.g., Air + Waste water = 2). Variables marked as descriptive have no assigned weight as they characterize the scenario but have no influence on the resulting concern score (*$h_{C, i}$ and $e_{C, i}$*). The variable Exposure prevention is, in this case, not applicable to ecosystem health.*

|  | **Human health exposure scoring (**$\boldsymbol{h}_{\boldsymbol{B, i}}$**),** $h_{B, i}=h_{V_{4,i}}\cdot h_{V_{5,i}}\cdot h_{{V_{6,i}}}\cdot h_{{V_{7,i}}}\cdot h_{{V_{8,i}}}\cdot h_{{V_{9,i}}}$ | |  | **Ecosystem health exposure scoring (**$\boldsymbol{e}_{\boldsymbol{B, i}}$**),**  $e_{B, i}= e_{V_{4,i}}\cdot e_{V_{5, i}}\cdot e_{V_{6,i}}\cdot e_{V_{7,i}}\cdot e_{{V_{8,i}}}$ | |  |
| --- | --- | --- | --- | --- | --- | --- |
|  | ***Variable*** | **Options to describe variable** | ***Weight***  ***(pts)*** | ***Variable*** | **Options to describe variable** | ***Weight***  ***(pts)*** |
| **Exposure description variables** | ***(***$\boldsymbol{h}_{\boldsymbol{V}_{\boldsymbol{4,i}}}$***)***  ***Life cycle stage***  *(descriptive)* | Life cycle stage and unit process in which the scenario occurs |  | ***(***$\boldsymbol{e}_{\boldsymbol{V}_{\boldsymbol{4,i}}}$***)***  ***Life cycle stage***  *(descriptive)* | Life cycle stage and unit process in which the scenario occurs |  |
|  | ***(***$\boldsymbol{h}_{\boldsymbol{V}_{\boldsymbol{5,i}}}$***)***  ***Exposure point*** *(descriptive)* | Point in which a population comes in contact with the substance. Air, wastewater, surface water, soil, dust, direct contact, (ingestion or dermal) |  | **(**$\boldsymbol{e}_{\boldsymbol{V}_{\boldsymbol{5,i}}}$**)**  ***Exposure point.***  *Several can be selected. The weight increases according to the number of selected points* *(accumulative)* | A. Air | +1 |
|  |  |  |  |  | B. Wastewater | +1 |
|  |  |  |  |  | C. Surface water | +1 |
|  |  |  |  |  | D. Soil | +1 |
|  |  |  |  |  | E. Dust | +1 |
|  | ***(***$\boldsymbol{h}_{\boldsymbol{V}_{\boldsymbol{6,i}}}$***)***  ***Exposure prevention*** | A. Not in place | 1 |  | | |
|  |  | B. In place and exposure is reduced | 0.5 |  |  |  |
|  |  | C. In place and exposure is eliminated | 0 |  |  |  |
|  | ***(***$\boldsymbol{h}_{\boldsymbol{V}_{\boldsymbol{7,i}}}$***)***  ***Frequency of exposure*** | A. Chronic | 2.5 | ***(***$\boldsymbol{e}_{\boldsymbol{V}_{\boldsymbol{6,i}}}$***)***  ***Frequency of exposure*** | A. Chronic | 2.5 |
|  |  | B. Repeated | 2 |  | B. Repeated | 2 |
|  |  | C. Single | 1.5 |  | C. Single | 1.5 |
| **Emission description variables** | ***(***$\boldsymbol{h}_{\boldsymbol{V}_{\boldsymbol{8,i}}}$***)***  ***Emission volume*** *(According to risk management measures that reduce emissions)* | A. No control mechanism in place - emissions occur | 1 | ***(***$\boldsymbol{e}_{\boldsymbol{V}_{\boldsymbol{7,i}}}$***)***  ***Emission volume*** *(According to risk management measures that reduce emissions)* | A. No control mechanism in place - emissions occur | 1 |
|  |  | B. Control mechanism in place – reduced emissions | 0.5 |  | B. Control mechanism in place – reduced emissions | 0.5 |
|  |  | C. Control mechanism in place – no emissions occur | 0 |  | C. Control mechanism in place – no emissions occur | 0 |
|  |  | D. Known concentrations and/or emissions remain below threshold values | 0 |  | D. Known concentrations and/or emissions remain below threshold values | 0 |
|  | ***(***$\boldsymbol{h}_{\boldsymbol{V}_{\boldsymbol{9,i}}}$***)***  ***Aggravating conditions*** | A. No aggravating conditions | 1 | ***(***$\boldsymbol{e}_{\boldsymbol{V}_{\boldsymbol{8,i}}}$***)***  ***Aggravating conditions*** | A. No aggravating conditions | 1 |
|  |  | B. Aggravating conditions that have a small effect on emissions | 1.5 |  | B. Aggravating conditions that have a small effect on emissions | 1.5 |
|  |  | C. Aggravating conditions that have a large effect on emissions | 2 |  | C. Aggravating conditions that have a large effect on emissions | 2 |

**Effect scoring variables (**$\boldsymbol{h}_{\boldsymbol{A, i}}$***and*** $\boldsymbol{e}_{\boldsymbol{A, i}}$**) (Table B1)
(**$\boldsymbol{h}_{{\boldsymbol{V}_{\boldsymbol{1,i}}}}$ **and** $\boldsymbol{e}_{{\boldsymbol{V}_{\boldsymbol{1,i}}}}$**) Hazard category** – The options are based on Annex VI of the CLP Regulation (European Commission, 2009), covering human and ecosystem health hazards. Following ECHA Guidance (European Chemicals Agency, 2011), systemic human health effects (CMR) receive higher scores than local effects (e.g. skin corrosion). For ecosystems, aquatic toxicity is scored higher than ozone layer damage, reflecting exceedance of planetary boundaries (Persson et al., 2022; Rockström et al., 2009; Steffen et al., 2015).

**(**$\boldsymbol{h}_{{\boldsymbol{V}_{\boldsymbol{2,i}}}}$ **and** $\boldsymbol{e}_{{\boldsymbol{V}_{\boldsymbol{2,i}}}}$**) Persistence, mobility, and bioaccumulation** – Developers can indicate if the substance they are analyzing presents any of the characteristics. The variable is applicable to both human and ecosystem health, and the provided options are based on the CLP Regulation, (European Commission, 2009). Under ECHA Guidance (European Chemicals Agency, 2014), vPvB/vPvM substances are most problematic, therefore “very” persistent/bioaccumulating scores higher than persistent/bioaccumulating.

**(**$\boldsymbol{h}_{{\boldsymbol{V}_{\boldsymbol{3,i}}}}$ **and** $\boldsymbol{e}_{{\boldsymbol{V}_{\boldsymbol{3,i}}}}$**) Exposed group and ecosystem vulnerability** - We defined the groups to provide developers with options to describe scenarios that are relevant to their application in a particular unit process. In the case of human health, distinctions are made between workers and the general population to describe during production related phases, and between general and vulnerable populations for the use phase. This enables the identification of at-risk groups and to target risk mitigation strategies. In the case of ecosystem health, distinctions are made between ecosystem and vulnerable ecosystems.

**Exposure scoring variables (**$\boldsymbol{h}_{\boldsymbol{B, i}}$***and*** $\boldsymbol{e}_{\boldsymbol{B, i}}$**) (Table B2)
(**$\boldsymbol{h}_{{\boldsymbol{V}_{\boldsymbol{4,i}}}}$ **and** $\boldsymbol{e}_{{\boldsymbol{V}_{\boldsymbol{4,i}}}}$**) Life cycle stage and unit process** – This is a descriptive variable and is assigned no scores. Developers can specify life cycle stage and unit process in which the scenario occurs.

**(**$\boldsymbol{h}_{{\boldsymbol{V}_{\boldsymbol{5,i}}}}$ **and** $\boldsymbol{e}_{{\boldsymbol{V}_{\boldsymbol{5,i}}}}$**) Exposure point** – The variable describes compartments where emissions occur. For human health, developers can specify how populations may be exposed (air, water, dust, or direct contact (i.e., ingestion or dermal contact)), This allows the description of a wide variety of products and potential exposure scenarios. For ecosystems, all possible exposure points must be indicated; these are aggregated into a multiplier for the exposure scenario score ($e_{B, i}$), for example if a substance is found to be present in soil, water, and air then eV5 = 3. This is meant to reflect increased exposure via multiple fate/pathways.

**(**$\boldsymbol{h}_{{\boldsymbol{V}_{\boldsymbol{6,i}}}}$**) Exposure prevention** – The variable applies only to human health. Developers the presence of indicate risk management measures (e.g., personal protection, ventilation, product design). Absence of measures leaves the score unchanged; presence reduces or nullifies the score depending on effectiveness.

**(**$\boldsymbol{h}_{{\boldsymbol{V}_{\boldsymbol{7,i}}}}$ **and** $\boldsymbol{e}_{{\boldsymbol{V}_{\boldsymbol{6,i}}}}$**) Frequency of exposure** – The variable is relevant for both human and ecosystem health. Differentiates scenarios where exposure to the substance is a single occurrence, or when exposure occurs more regularly. In the concern score system, chronic exposure is considered more problematic, increasing the resulting score, since it can lead to prolonged effects for some substances.

**(**$\boldsymbol{h}_{{\boldsymbol{V}_{\boldsymbol{8,i}}}}$ **and** $\boldsymbol{e}_{{\boldsymbol{V}_{\boldsymbol{7,i}}}}$**) Emission volume** - The variable is relevant for both human and ecosystem health. For the purposes of the tool any exposure scenario implies emissions. The options allow the description of emission volumes based on available information on concentration, threshold data, or risk management presence. Emissions below threshold or eliminated by controls nullify the score; absence of controls leaves the score unchanged; partial controls halve it. This enables the comparison of design interventions.

**(**$\boldsymbol{h}_{{\boldsymbol{V}_{\boldsymbol{9,i}}}}$ **and** $\boldsymbol{e}_{{\boldsymbol{V}_{\boldsymbol{8,i}}}}$**) Aggravating conditions** - The variable is relevant for both human and ecosystem health. Developers describe specific conditions that the product may be exposed to, that may intensify emissions (e.g., temperature, humidity, wear, machinery, maintenance, shredding, substance properties such as solubility, volatility, adsorption). The options are absent, which leaves the score unchanged; slight aggravation, which increases the concern score; and clear aggravation, which doubles the concern score. The assessment of aggravating conditions is qualitative, aiming to inform mitigation strategies.

## 3. Uncertainty score

As the tool is applied by users during the early stages of the product development process, a relatively high level of uncertainty is inherent. To address this, an Uncertainty Score ($h_{U, i}$ for human health scenarios or $e_{U, i}$ for ecosystem health scenarios) (highlighted in blue in Figure B3) is calculated for each exposure scenario, complementing its Concern Score ($h_{C, i}$ and $e_{C, i}$). This helps indicate data gaps, while allowing flexibility when data is limited, inconclusive, or unavailable. When assessing each exposure scenario and selecting from the variable options (Tables B1 and B2), the developer can also indicate the level of uncertainty of their answer for each variable. Each indication of uncertainty has a predefined weight (points), as shown below in Table B3. For example, if variable $e_{V_{6,i}}$ *Frequency of exposure* is marked as chronic, but the developer is not entirely sure the data point is correct, then its uncertainty weight can be marked as $U_{e_{V_{6,i}}}$ = 2pts (medium uncertainty).

*Table B3. Weights assigned to indicate the level of uncertainty for each variable (e.g.,* $h_{V_{1,i}}$*,* $e_{V_{1,i}}$*, etc.). These weights are used to calculate the overall uncertainty score for each exposure scenario.*

| ***Level of uncertainty*** | ***Description*** | ***Weight (pts)*** |
| --- | --- | --- |
| No uncertainty | The data point is certain (within 10%) | 1 |
| Low uncertainty | The data point is a reasonable estimation based on other available data (10-50%) | 1.5 |
| Medium uncertainty | The data point is not a reliable estimation or comes from an unreliable source, and requires further research (50-200%) | 2 |
| High uncertainty | The data point is a guess to illustrate a scenario but requires further research (>200%) | 3 |

To calculate the uncertainty score ($h_{U, i}$ or $e_{U, i}$) for the analyzed exposure scenario, the tool multiplies the uncertainty levels (Table 3) indicated for each variable (Tables 1 and 2):

$$\begin{aligned} h_{U, i}= U_{h_{V_{1,i}}} \cdot U_{h_{V_{2,i}}} \cdot U_{h_{V_{3,i}}} \cdot U_{h_{V_{4,i}}} \cdot U_{h_{V_{5,i}}} \cdot U_{h_{V_{6,i}}} \cdot U_{h_{V_{7,i}}} \cdot U_{h_{V_{8,i}}} \cdot U_{h_{V_{9,i}}}\#\left( 3 \right) \end{aligned}$$

And

$$\begin{aligned} e_{U, i}= U_{e_{V_{1,i}}} \cdot U_{e_{V_{2,i}}} \cdot U_{e_{\begin{aligned} V_{3,i} \\ \end{aligned}}} \cdot U_{e_{V_{4,i}}} \cdot U_{e_{V_{5,i}}} \cdot U_{e_{V_{6,i}}} \cdot U_{e_{V_{7,i}}} \cdot U_{e_{V_{8,i}}}\#\left( 4 \right) \end{aligned}$$

A higher uncertainty score indicates that the concern score is inconclusive and requires further research, either by gathering additional data for the specific scenario or by identifying potential hotspots to be addressed through preventive design strategies. Product developers receive an interpretation guide to support the simultaneous assessment of concern and uncertainty scores. This interpretation guide is included in the *MAPSSS tool protocol and steps*, available as Supplementary Material D.

***Visualizing and interpreting concern and uncertainty scores***

For each exposure scenario, a concern score ($h_{C, i}$ and $e_{C, i}$) and an uncertainty score ($h_{U, i}$ and $e_{U, i}$) are calculated, reflecting the potential risk level and the confidence in the underlying data. As numerous scenarios may be assessed and the individual scores are not intended for quantitative interpretation, the tool consolidates the results into a three-tier scale (low, medium, high) indicating the overall level of concern. This supports the identification of risk hotspots, data gaps, and comparisons between reference products, alternatives, or new designs. The results can be visualized next to each exposure scenario, as indicated in Figure B2. The concern scores appear as small, medium, or large filled pink triangles, and uncertainty scores as open blue triangles of corresponding sizes (Figure B1).

Product developers can use the following guidelines to interpret the maps and identify next steps:

- Prioritize high-scoring scenarios and assess their relevance to the product design; some may fall under the responsibility of other stakeholders, such as suppliers.
- Analyze score origins by reviewing the selected options to describe variables. If high scores result from aggravating conditions, preventing these may be a suitable strategy.
- Interpret combinations of concern and uncertainty scores:
  - Low concern – low uncertainty – The information is sufficient, and the scenario can be considered of low concern.
  - High concern – low uncertainty – The scenario represents a clear hotspot requiring mitigation through alternatives.
  - High concern – high uncertainty – Additional information is needed to confirm the concern; preventive measures should be considered.
  - Low concern – high uncertainty – The scenario demands caution. The low concern can be misleading and a result of lack of data or an underestimation of risks. Therefore, additional information is likely necessary and preventive measures should be considered.

## 4. References

Boersema, J. J., & Reijnders, L. (2009). Principles of environmental sciences. In *Principles of Environmental Sciences*. Springer Netherlands. https://doi.org/10.1007/978-1-4020-9158-2/COVER

Bolaños Arriola, J., Subramanian, V., Bakker, C., & Balkenende, R. (2023). Safe and Circular Design – A design method for dealing with substances of concern in products. In K. Niinimäki & K. Cura (Eds.), *5th PLATE Conference Proceedings 2023*. https://kth.diva-portal.org/smash/get/diva2:1795825/FULLTEXT01.pdf

European Chemicals Agency. (2011). *Guidance on information requirements and chemical safety assessment Part B: Hazard assessment Version 2.1*. http://echa.europa.eu/

European Chemicals Agency. (2014). *Guidance on information requirements and chemical safety assessment Chapter R11 : PBT/vPvB assessment* (Issue 2, pp. 1–119). http://echa.europa.eu/documents/10162/13559/mb_63_2013_consultation_procedure_for_gui

European Commission. (2009). *Classification, labelling and packaging of substances and mixtures, amending and repealing Directives 67/548/EEC and 1999/45/EC, and amending Regulation (EC) No 1907/2006*. https://www.echa.europa.eu/regulations/clp/legislation

Guinée, J. B., Heijungs, R., Vijver, M. G., & Peijnenburg, W. J. G. M. (2017). Setting the stage for debating the roles of risk assessment and life-cycle assessment of engineered nanomaterials. *Nature Nanotechnology*, *12*(8), 727–733. https://doi.org/10.1038/nnano.2017.135

Persson, L., Carney Almroth, B. M., Collins, C. D., Cornell, S., de Wit, C. A., Diamond, M. L., Fantke, P., Hassellöv, M., MacLeod, M., Ryberg, M. W., Søgaard Jørgensen, P., Villarrubia-Gómez, P., Wang, Z., & Hauschild, M. Z. (2022). Outside the Safe Operating Space of the Planetary Boundary for Novel Entities. *Environmental Science and Technology*, *56*(3), 1510–1521. https://doi.org/10.1021/ACS.EST.1C04158/ASSET/IMAGES/LARGE/ES1C04158_0002.JPEG

Rockström, J., Steffen, W. L., Noone, K., Persson, Å., Stuart, F., Iii, C., Rockstrom, J., Steffen, W., Noone, K., Persson, A., Chapin, F. S., Lambin, E., Lenton, T. M., Scheffer, M., Folke, C., Schellnhuber, H., Nykvist, B., De Wit, C. A., Hughes, T., … Foley, J. (2009). *Planetary Boundaries: Exploring the Safe Operating Planetary Boundaries: Exploring the Safe Operating Space for Humanity Space for Humanity Citation Details Citation Details*.

Steffen, W., Richardson, K., Rockström, J., Cornell, S. E., Fetzer, I., Bennett, E. M., Biggs, R., Carpenter, S. R., De Vries, W., De Wit, C. A., Folke, C., Gerten, D., Heinke, J., Mace, G. M., Persson, L. M., Ramanathan, V., Reyers, B., & Sörlin, S. (2015). Planetary boundaries: Guiding human development on a changing planet. *Science*, *347*(6223). https://doi.org/10.1126/SCIENCE.1259855/SUPPL_FILE/STEFFEN-SM.PDF

Swedish Chemical Agency, K. (2020). *Hazard and risk assessment of chemicals-an introduction*. www.kemi.se/en/guidance-on-national-chemicals-control

Van Leeuwen, C. J., & Vermeire, T. G. (2007). Risk Assessment of Chemicals: An Introduction. In C. J. van Leeuwen & T. G. Vermeire (Eds.), *American Journal of Therapeutics* (Issue 3). Springer Netherlands. https://doi.org/10.1007/978-1-4020-6102-8
