# Supplementary material for: Mapping Uncertain Risks and Impacts of Substances of Concern in Product Life Cycles - An SSbD Tool for Product Developers
Source: Circ Econ Sustain. 2026 Aug 3;6(4):372. doi: 10.1007/s43615-026-01077-w (PMC13433538; doi:10.1007/s43615-026-01077-w)
Supplement: Supplementary file 3 [file 43615_2026_1077_MOESM4_ESM.docx]

**Supplementary material D – MAPSSS tool protocol and steps**

This supplementary material includes the detailed description of the proposed steps for developers to use the Mapping Assessment for Product Substance Safety and Sustainability (MAPSSS) tool.

Table of Contents

[1.	Introduction and scope of the tool	1](#_Toc212817349)

[2.	Stage 1 - Mapping and Risk Assessment of the existing product	2](#_Toc212817350)

[1.	Defining a Functional Unit and Reference Flows	3](#_Toc212817351)

[2.	System mapping – Unit processes	3](#_Toc212817352)

[3.	System mapping – Inputs, outputs, and the presence of the SoC in the lifecycle	4](#_Toc212817353)

[4.	Mapping of exposure scenarios	5](#_Toc212817354)

[5.	Assessment of substance risks – Calculating and mapping the concern score	7](#_Toc212817355)

[3.	Stage 2 – Risk and tradeoff assessment of new design(s) and alternatives	10](#_Toc212817356)

[4.	Stage 3 - Comparison of maps of existing product and new design(s) RA + LCA	13](#_Toc212817357)

## 1. Introduction and scope of the tool

The *Mapping Assessment for Product Substance Safety and Sustainability (MAPSSS)* is targeted at product developers to support them in dealing with the risks generated by substances of concern (SoC) in products. It is an assessment and visualization tool that integrates basic principles of two well established assessment methods, chemical risk assessment (RA), and life cycle assessment (LCA). The tool aims to provide indicators that highlight risk hotspots and support decision making, especially in early stages of the development process with high uncertainty. Figure D1 provides an overview of the three main stages and steps proposed for developers to integrate the tool elements into the design process. Each of the tool stages contains a set of steps that correspond to the main goal or activity of the design process phase they belong to. The mapping tool supports developers to:

1. Identify and visualize risks of known hazardous chemicals and materials throughout the lifecycle and consecutive lifecycles of the reference product.
2. Prioritize and identify impact hotspots in the lifecycle and consecutive lifecycles of the reference product.
3. Compare alternatives meant to mitigate the identified risks. The method affords the assessment and comparison of interventions at different levels, such as alternative chemicals or materials, or alterations to the product design, following the strategies proposed by (Bolaños Arriola et al., 2024): Avoid, Reduce, Control. The comparison considers safety and potential environmental trade-offs of alternatives.
4. Highlight uncertainties in the assessment of the reference product and its alternatives.

**Overview of process and steps**

Prior to the use of MAPSSS, designers should define the goal and scope of the study, similar to established LCA protocols (e.g., Guinée et al. [30]). In this stage, the designer can define for instance, the geographic and temporal coverage of the analysis, as well as defining the goal, for instance, the identification of risk hotspots in an existing product, the identification of specific knowledge gaps, or the comparison of alternatives. When developing the system map, the designer can also establish which unit processes are to be analyzed and which fall out of the scope of the analysis.

Figure D1 provides an overview of the three main stages of the tool and how it integrates into the design process.

Stage 1 - Mapping and Risk Assessment of the reference product

During this stage, developers map and assess the SoC risks for the reference product or design, using the mapping elements, concern and uncertainty score calculators, and interpretation guidelines. This takes place in the Discover and Define phases, requiring several iterations to obtain all the necessary information to perform the assessment and arrive at a problem definition. It is assumed that the product developer is aware of or suspecting the presence of an SoC in the reference product before commencing Stage 1. Stage 1 concludes with the identification of substance risks and uncertainty hotspots in the reference product.

Stage 2 - Mapping and Risk Assessment of new design(s)
After developing concepts that address the risk hotspots identified during stage 1, developers can map and assess the substance risks of these new concepts or designs during stage 2. An assessment must be performed for each concept, following the same set of steps used to assess the reference product. During this stage, developers also perform a qualitative analysis to identify potential environmental tradeoffs resulting from the design changes by applying the axioms. This supports early identification of unintended environmental impacts. Stage 2 takes place during the Development phase of the design process.

Stage 3 - Comparison of maps of existing product and new design(s)
After the assessments of the reference product and new concepts, developers can proceed to compare the resulting maps. Stage 3 provides guidance to compare in terms of risk mitigation, potential environmental tradeoffs, and uncertainty. This stage takes place late in the Development phase of the design process and should provide designers with information for comparison of concepts and decision-making. Stage 3 should also provide uncertainty indicators that should be addressed throughout the Deliver phase of the process.

*Figure D1. Stages, steps, and integration of the MAPSSS tool into the design process. The level of knowledge of the analyzed product is represented by the circle filling in as the process progresses. The other geometrical figures represent new designs or alternatives.*

## 2. Stage 1 - Mapping and Risk Assessment of the existing product

The main goal of stage 1 is to assess the substance(s) risks of the reference product or design, to identify substance(s) risk hotspots, and uncertainty. The results obtained from this stage can be used as a reference for ideation, to mitigate the substance risks. This stage should be followed iteratively to detail the necessary information to analyze and map all exposure scenarios throughout the lifecycle of the product, employing for example, supply chain research. A template and mapping elements are provided through the Miro platform (Figures D2 and D3). The template is available in here: <https://miro.com/app/board/uXjVJxz0NLE=/?share_link_id=754270951757>

### 1. Defining a Functional Unit and Reference Flows

- Define the function, which is the service provided by a product or process, e.g., charging a phone, illuminating a room, providing protection from the rain.
- Define the functional unit, which is the quantified form of the function provided by the product system, e.g., charging a phone for 3 years or 1000 charging cycles, or illuminating a room for 500 hours at 700 lumens. The functional unit for the mattress case was defined as: *Providing pressure-relieving support for hospital patients for 5 years, under typical clinical use conditions, 24-hour use, with an average of 1 patient/day and cleaning between patients, with a standard size mattress that meets flammability norms*
- The functional unit should be the same for the original product and all alternative concepts, to allow their comparison. Using the functional unit, define the reference flow, which represents one way to obtain the functional unit. The reference flow = functional unit (FU) + specific alternative, e.g., in the case of the mattress, the reference flow for the reference product was defined as: *Providing pressure-relieving support for hospital patients for 5 years, under typical clinical use conditions, 24-hour use, with an average of 1 patient/day and cleaning between patients, with a standard size polyurethane foam mattress with flame retardants that meets flammability norms (Figure D7).* The reference flow for one of the alternatives was defined as: *Providing pressure-relieving support for hospital patients for 5 years, under typical clinical use conditions, 24-hour use, with an average of 1 patient/day and cleaning between patients, with a standard size polyester core mattress that meets flammability norms (Figure D8).*
- Fill in the reference flow for the reference product in the system map template in the Miro board (Figure D2). You can generate reference flows for the alternative designs in Stage 2.

### 2. System mapping – Unit processes

- The system template (Figure D2) predefines the lifecycle stages in which you can map the relevant unit processes (Figure D3). The lifecycle is structured into the following stages:
  • Chemical production: all processes required to produce the analyzed substance and other relevant substances in materials.
  • Product manufacturing: material production, processing, and assembly.
  • Retail: processes required to deliver the product to its use.
  • Use: activities related to product operation and maintenance, including potential misuse.
  • End-of-life: processes to destroy, repurpose, or reprocess the used product.
- Map all unit processes involved your product system in the different lifecycle stages. Use the system map template (Figure D2) and mapping elements (Figure D3) available in the Miro. Follow the convention shown in Figure D5.
- Consider single or grouped unit operations in the system for which inputs and outputs can be estimated. Whenever possible, consider the smallest element, as this will provide a higher level of detail to your assessment. For example, instead of considering “mattress production” as a single unit process, you should break it into smaller units i.e., “foam production”, “coating production”, “assembly”, etc.
- When mapping unit processes always consider the specific contextual conditions that apply to the product you are assessing. Consider for example, the geographical context.
- For the use phase consider all different possible use and misuse scenarios. Considering who might be involved and the conditions to which the product is exposed. For example, “mattress cleaning”, “mattress storage”.
- For EoL consider the scenario or scenarios that apply to the contextual situation the product system operates in, e.g., recycling, incineration, landfill, etc. Consider the potential multiple processes involved in circular scenarios for example, remanufacturing, repair, refurbishing, etc. Consider as well undesirable EoL scenarios.

### 3. System mapping – Inputs, outputs, and the presence of the SoC in the lifecycle

- Map all inputs and outputs of each unit process in your system. For example, in Figure D7, the unit process *mattress assembly* has a mattress core, a mattress cover, and energy as inputs, and generates a mattress as an output.
- Inputs and outputs are represented horizontally in the map. Use the mapping elements in the convention indicated in Figure D5.
- Use the Bill of Materials (BOM) and material composition information from suppliers as a starting point to obtain the necessary information.
- Whenever possible indicate material mass and processing energy. However, it is possible to map the system with less accuracy in initial stages and detail the information in later iterations when more information becomes available. Initial, low detail maps are useful to highlight uncertainty and knowledge gaps.
- Indicate all transportation as inputs and not as unit processes.
- Map any inputs/outputs that represent the circularity of the system if applicable. For example, the output of a recycling process, can be mapped as an input in a production process, indicating circularity.
- Outputs can also be mapped outside the system boundary. In the case of the mattress for example (Figure D7), the waste generated during the mattress cutting process is mapped as an output leaving the system boundary, as the fate of this residual material may be unknown.
- Once all inputs and outputs are mapped, map the presence of SoC across the product life cycle by placing rhomboid markers (Figure D3 and D5) on the inputs and outputs where the SoC is expected to be present. This indicates the behavior of the SoC and identifies “contaminated” inputs and outputs. In products where circular economy strategies are present, potentially contaminated outputs can flow back into the system (in case of closed loop circularity) or out of the system (in case of open loop circularity). In addition, these indicators flag potential risks from outputs crossing the system boundary, since their fate is unknown and their impacts can therefore not be assessed.
- The pink (filled) marker identifies substances that are known as SoCs. The blue (outline) marker highlights substances with unknown or inconclusive safety profiles. Figure D5 shows an example of the proposed convention to use the SoC indicators.

*Figure D2. The life cycle template with five predefined lifecycle stages. The template serves as a basis for system and exposure scenario mapping and presents the reference flow of the analyzed product or alternative.*

*Figure D3. Visual elements for system mapping, indicators for “contaminated” inputs and outputs, and indicators for potential environmental trade-offs.*

### 4. Mapping of exposure scenarios

- Identify and describe all applicable exposure scenarios for each unit process using the mapping elements provided in Figure D4 using the convention indicated in Figure D5. All mapping elements are available in the Miro board.
- You can indicate exposure scenarios using three variables: whether humans and/or ecosystems are impacted, the points at which exposure can occur (e.g., soil, water), and the exposed population (e.g., occupational, bystanders, vulnerable ecosystems).
- You can define multiple exposure scenarios per unit process as indicated in the example in Figure D5. For human health, a scenario is created for each exposed group of people; for ecosystem health, one scenario per unit process is considered. The example in Figure D5 shows three scenarios: two for human health (both via air, but affecting two different groups: bystanders and occupational) and one for ecosystem health (via water and soil).
- Add an additional specification (pink asterisk) if aggravating conditions are suspected, e.g., temperature or humidity variation, wear and tear from behavior or machinery, or interaction with other substances during maintenance. Assess whether design-specific conditions (e.g., use at high temperatures, shredding at end of life) combined with intrinsic substance characteristics (e.g., solubility, volatility, adsorption) may aggravate emissions.
- While defining exposure scenarios, consider a variety of product specific scenarios, including relevant stakeholders, and other aspects such as contextual factors, and behavior.

*Figure D4. Visual elements to map and describe exposure scenarios, indicating whether human health or environmental health is affected (scenario type), the exposed groups (exposed population or ecosystem vulnerability), exposure points, potential presence of aggravating conditions (e.g., heat, misuse, etc.), and resulting concern and uncertainty scores.*

Figure D5. *Proposed convention to apply the tool’s mapping elements. The example depicts a unit process with a contaminated input and output, and describes three exposure scenarios, two for human health via air to bystanders and occupational, and one for ecosystem health via water and soil. Aggravating conditions appear to be present for the occupational exposure scenario.*

### 5. Assessment of substance risks – Calculating and mapping the concern score

- You can assess and score the exposure scenarios defined on the map to identify hotspots within the product life cycle. To do this, use the concern and uncertainty score calculator, which is a methodological adaptation and simplification of the RA framework. The calculator is available in Supplementary Material C.
- Calculate a concern ($h_{C, i}$ = *human health* and $e_{C, i}$= *ecosystem health*) and uncertainty score ($h_{U, i}$ = *human health* and $e_{U, i}$ = *ecosystem health*) for each of the established exposure scenarios in step 4 (note that *i* is representative of a SoC). Use the excel concern score calculator (Figure D6). The excel form provides you with drop down menus to choose the option that best describes a variable, indicating substance characteristics and behavior. Only fill up information on the cells marked in blue. Be aware that the excel differentiates between human health and ecosystem health scenarios.
- After assessing all scenarios, you will obtain a set of results in a 3-color scale (light, medium, dark). These indicate low, medium, or high concern (red) and uncertainty (blue) scores respectively. Keep in mind that the individual scores in the excel are not intended for quantitative interpretation.
- Once you obtain the scores using the excel template, indicate the concern score in the system map using the mapping elements (Figure D4) in the convention indicated in Figure D5. The concern scores should be indicated as small, medium, or large filled pink triangles, and uncertainty scores as open blue triangles of corresponding sizes (Figure 4).
- Use the following guidelines to interpret the maps and identify next steps:
  - Prioritize high-scoring scenarios and assess their relevance to the product design; some may fall under the responsibility of other stakeholders, such as suppliers.
  - Analyze score origins (excel) by reviewing the selected options to describe variables. If high scores result from aggravating conditions, preventing these may be a suitable strategy.
  - Interpret combinations of concern and uncertainty scores:
    - Low concern – low uncertainty – The information is sufficient, and the scenario can be considered of low concern.
    - High concern – low uncertainty – The scenario represents a clear hotspot requiring mitigation through alternatives.
    - High concern – high uncertainty – Additional information is needed to confirm the concern; preventive measures should be considered.
    - Low concern – high uncertainty – The scenario demands caution. The low concern can be misleading and a result of lack of data or an underestimation of risks. Therefore, additional information is likely necessary and preventive measures should be considered.

*Figure D6. Excel template to calculate concern and uncertainty scores.*

*Figure D7. Resulting map of the reference hospital mattress containing TCPP as a flame retardant. The map illustrates the use of the tool elements, including system mapping of the product, indicators of contaminated inputs and outputs, exposure scenarios for human and environmental health, results for concern and uncertainty scores, and aggravating conditions.*

## 3. Stage 2 – Risk and tradeoff assessment of new design(s) and alternatives

### 1. Risk mapping assessment of alternatives

- Make a map and assessment for EACH new design or alternative following the SAME set of steps you followed to analyze the reference product in stage 1.
- Maintain the same Functional Unit and only adapt the reference flow accordingly. The reference flow for one of the alternatives was defined as: *Providing pressure-relieving support for hospital patients for 5 years, under typical clinical use conditions, 24-hour use, with an average of 1 patient/day and cleaning between patients, with a standard size polyester core mattress that meets flammability norms (Figure D8).*
- When mapping the product system (unit processes, inputs, outputs, etc.), as much as possible, maintain the same level of detail in comparison to the initial map (Stage 1).

### 2. Identifying potential environmental tradeoffs

- Once all alternatives or new designs have been mapped, you can perform an analysis to identify potential environmental tradeoffs.
- Perform a qualitative analysis to identify potential environmental trade-offs from design changes or mitigation strategies addressing the SoC. Use the axiom list in Table D1 to reflect on each alternative or new design. Mark any potential tradeoffs on the system maps of each of the alternatives by highlighting the relevant unit process in green as indicated in Figure D3.
- To perform the assessment, you can consult sources such as bills of materials, basic (open source) LCI databases, analysis of user behavior, or product performance tests. For example, Figure D8 shows that potential environmental trade-offs must be considered due to the change in core material and the need for additional cleaning and maintenance processes.

*Table D1. Axioms for potential environmental impact trade-offs*

| **Production** | **Transport** | **Use** | **EoL** |
| --- | --- | --- | --- |
| **The extraction of the substitute material or additive is more impactful**  (Meng et al., 2024)  *(e.g., stainless-steel container instead of plastic with additives that are SoC)* | **Transportation impact is increased due to increase in mass, variety of materials, supplier location, etc.**  (Bolaños Arriola et al., 2024; Kravchenko et al., 2020)  *(e.g., longer distances as a consequence of substitution, substitute for PVC cables is denser and requires a thicker layer)* | **The durability is reduced necessitating earlier replacement**  (Bolaños Arriola et al., 2024; Meng et al., 2024)  *(e.g. substituting plastics with materials like glass or metal resulted in higher overall environmental impacts, partly due to increased material fragility, substitute additives in PVC cables reduce durability)* | **New processing steps or infrastructure are necessary**  (Bolaños Arriola et al., 2024; Fenwick et al., 2023)  *(e.g. reducing SoC impacts by increasing useful life in electronics through applying composite materials can require additional steps like cleaning, sorting, and material separation during EoL processing)* |
| **Processing is more complex or requires more steps and/or is energy intensive**  (Bolaños Arriola et al., 2024; Kravchenko et al., 2020)  *(e.g. Reduction of VOC content in chemicals complicates the processing of the chemical, higher production costs and changes in the production methods are a consequence of substitute blowing agents)* |  | **The alternative requires maintenance or repairs**  (Bolaños Arriola et al., 2024; Sandionigi, 2024)  **(***e.g. material efficiency in electronics vs reliability and safety, increasing the useful life of products can require additional maintenance and repair)* | **Processing waste/resources is more energy intensive**  (Meng et al., 2024)  *(e.g. recycling aluminum is more impactful than microplastic generating PET)* |
| **More material is necessary to meet a function, due to performance, or to prevent risk**  (Bolaños Arriola et al., 2024; Kravchenko et al., 2020)  *(e.g. small amounts of a toxic material vs larger amount of a less toxic material, additional components to prevent risk)* |  | **The alternative has higher energy consumption / reduced efficiency / more waste generated**  (Bolaños Arriola et al., 2024; Byggeth & Hochschorner, 2006; Luttropp & Lagerstedt, 2006)  *(e.g****.*** *substitute refrigerant gases can have consequences on the appliance performance and energy consumption)* | **Processing techniques are not available (close by)**  (Beames et al., 2021)  *(e.g. disassembly of mattresses needs large ovens to melt adhesive which are only available in specific areas, additional logistical challenges)* |
| **‘Known’ less sustainable material vs ‘unknown’ potentially more sustainable material**  (Bolaños Arriola et al., 2024; Kravchenko et al., 2020)  *(e.g., Substitution of PFAS with non-fluorinated substances, often with an unknown safety profile)* |  |  | **The new material/product/chemical hinders circularity**  (Bolaños Arriola et al., 2024; Thompson et al., 2020)  *(e.g.* *Containment of components for safety inhibits disassembly, material is not recyclable)* |
| **New impacts when transitioning to a service/digital product**  (Bolaños Arriola et al., 2024; Chen & Huang, 2019)  *(e.g., additional infrastructure is necessary to meet afunction)* |  |  |  |

*Figure D8. Resulting map of the alternative hospital mattress with a polyester labyrinth core. The map illustrates the use of the tool elements, including system mapping of the product, indicators of contaminated inputs and outputs, exposure scenarios for human and environmental health, results for concern and uncertainty scores, and aggravating conditions. Potential environmental trade-offs are indicated by unit processes highlighted in green.*

## 4. Stage 3 - Comparison of maps of existing product and new design(s) RA + LCA

- A comparison of maps can be performed once you have mapped the reference product and one or more alternatives or new designs. The comparison analysis focuses on three aspects: 1) SoC safety, 2) risk tradeoffs (considering uncertainty), and 3) potential environmental tradeoffs, as defined by the tool development criteria in section 2.1.
- Follow these guidelines for analysis:
  1. Observe if any changes on “contaminated” inputs and outputs are present. Especial attention should be given to inputs and outputs marked with blue (outline) rhomboids, indicating substances with unknown or inconclusive safety profiles. Marked outputs that exit the system boundary or generate open, or closed loop (circularity) scenarios should be treated with caution.
  2. Compare all resulting concern and uncertainty scores. Numerical results from the concern score calculator should not be used quantitatively as this is a semi-quantitative assessment. Focus should be in the reduction or elimination of risk hotspots, while preventing uncertainty hotspots. The creation of new risk scenarios should also be evaluated.
  3. Analyze the presence of potential environmental trade-offs. This streamlined form of LCA provides indicators to obtain further information, and help prevent unforeseen impacts.
- For example, Figure D8 indicates that while the polyester structure reduces SoC risks in comparison to the reference product (Figure D7), potential environmental trade-offs must be considered due to the change in core material and the need for additional cleaning and maintenance processes. Furthermore, the figure highlights a potential circular scenario where shredded foam re-enters the system, for which risks from recycled foam sources and other contaminants should be considered.
